# Analysis of the metabolic proteome of lung adenocarcinomas by reverse-phase protein arrays (RPPA) emphasizes mitochondria as targets for therapy

**DOI:** 10.1038/s41389-022-00400-y

**Published:** 2022-05-09

**Authors:** Laura Torresano, Fulvio Santacatterina, Sonia Domínguez-Zorita, Cristina Nuevo-Tapioles, Alfonso Núñez-Salgado, Pau B. Esparza-Moltó, Lucía González-Llorente, Inés Romero-Carramiñana, Cristina Núñez de Arenas, Brenda Sánchez-Garrido, Laura Nájera, Clara Salas, Mariano Provencio, José M. Cuezva

**Affiliations:** 1 Departamento de Biología Molecular, Universidad Autónoma de Madrid (UAM), 28049 Madrid, Spain; 2 Centro de Biología Molecular Severo Ochoa, Consejo Superior de Investigaciones Científicas-Universidad Autónoma de Madrid (CSIC-UAM), 28049 Madrid, Spain; 3 Centro de Investigación Biomédica en Red de Enfermedades Raras (CIBERER), Instituto de Salud Carlos III, ISCIII, 28029 Madrid, Spain; 4 Instituto de Investigación Sanitaria Hospital 12 de Octubre, Hospital 12 de Octubre, 28041 Madrid, Spain; 5 Servicio de Anatomía Patológica, Hospital Universitario Puerta de Hierro, Majadahonda, 28222 Madrid, Spain; 6 Oncología Médica, Hospital Universitario Puerta de Hierro, Majadahonda, 28222 Madrid, Spain

**Keywords:** Cancer metabolism, Targeted therapies, Prognostic markers, Predictive markers

## Abstract

Lung cancer is the leading cause of cancer-related death worldwide despite the success of therapies targeting oncogenic drivers and immune-checkpoint inhibitors. Although metabolic enzymes offer additional targets for therapy, the precise metabolic proteome of lung adenocarcinomas is unknown, hampering its clinical translation. Herein, we used Reverse Phase Protein Arrays to quantify the changes in enzymes of glycolysis, oxidation of pyruvate, fatty acid metabolism, oxidative phosphorylation, antioxidant response and protein oxidative damage in 128 tumors and paired non-tumor adjacent tissue of lung adenocarcinomas to profile the proteome of metabolism. Steady-state levels of mitochondrial proteins of fatty acid oxidation, oxidative phosphorylation and of the antioxidant response are independent predictors of survival and/or of disease recurrence in lung adenocarcinoma patients. Next, we addressed the mechanisms by which the overexpression of ATPase Inhibitory Factor 1, the physiological inhibitor of oxidative phosphorylation, which is an independent predictor of disease recurrence, prevents metastatic disease. We highlight that IF1 overexpression promotes a more vulnerable and less invasive phenotype in lung adenocarcinoma cells. Finally, and as proof of concept, the therapeutic potential of targeting fatty acid assimilation or oxidation in combination with an inhibitor of oxidative phosphorylation was studied in mice bearing lung adenocarcinomas. The results revealed that this therapeutic approach significantly extended the lifespan and provided better welfare to mice than cisplatin treatments, supporting mitochondrial activities as targets of therapy in lung adenocarcinoma patients.

## Introduction

Lung cancer is one of the most aggressive and lethal malignancies and the leading cause of cancer-related death worldwide [1]. Lung adenocarcinoma (LUAD) is the most common histological subtype of lung cancer, accounting for over 40% of non-small cell lung cancers (NSCLC) [2]. Large-scale genomic studies during the last decade have identified several driver genes [2, 3] and immune-checkpoint inhibitors (ICIs) of LUAD for a targeted therapy [2, 4, 5]. However, and despite the encouraging results obtained, the clinical benefit is limited because only a fraction of the patients has druggable gene alterations or recurrence of the disease inevitably occurs [6]. Hence, the incorporation of additional imaging [7, 8] and “omic” techniques aimed at profiling the dynamic nature of LUAD, their cellular heterogeneity, metabolome and proteome [9, 10] are required to develop an effective precision medicine for the patients.

Proteins control the metabolic rewiring experienced by tumors to account for their need of energy and metabolic precursors demanded by proliferation [11]. Hence, proteins involved in the aberrant activation of signaling pathways and in catalyzing the transformation of biomolecules offer potential biomarkers for a targeted therapy of LUAD [10, 12]. Mitochondria are key hubs controlling metabolism and cellular signaling, playing an important role in tumor progression and resistance to therapy [13, 14]. Recent preclinical studies have successfully targeted LUAD by exploiting their metabolic dependencies [15, 16], and targeting mitochondrial activities offers effective therapeutic strategies against cancer [9, 17–20]. However, the metabolic heterogeneity of LUAD and the variety of nutrients that can fuel tumor growth [21–23] make the development of targeted therapies more difficult. Moreover, a quantitative and integrated profile of the metabolic proteome of LUAD is missing [24], which is a required and committed step to identify the enzymes of metabolism that could be targeted to improve the therapeutic landscape of NSCLC. Reverse Phase Protein Arrays (RPPA) is a high-throughput quantitative immunological technique [25, 26] that has been implemented with success in profiling the aberrant activation of the signaling pathways in cancer and the mechanisms underlying sensitivity and resistance to cancer therapy [26, 27]. Hence, RPPA represent a powerful technique for the evaluation of the metabolic proteome in cancer with high clinical transposability.

Herein, we have followed a RPPA approach to quantitate the changes of twenty-seven proteins of metabolism in hundred and twenty-eight biopsies of tumor and paired non-tumor adjacent tissue (NAT) of LUAD patients to profile the changes in the metabolic proteome and to advance potential metabolic markers for a targeted therapy. We describe major increases in the content of the enzymes of glucose and fatty acid (FA) metabolism, oxidative phosphorylation (OXPHOS) and of the antioxidant response, as well as of biomarkers of protein oxidative damage in LUAD. Data transformation and classifier highlighted mitochondrial proteins as most relevant biomarkers of the metabolic signature of LUAD. Interestingly, the tumor content of a large number of the enzymes studied positively or negatively correlated with progression of the disease. Machine learning provided a signature of metastatic disease that further emphasized the relevance of biomarkers of OXPHOS and of the mitochondrial antioxidant response in LUAD progression and hence, as promising targets for an effective therapeutic strategy. Finally, we demonstrate in a preclinical mouse model bearing LUAD that the combination of drugs that target mitochondrial OXPHOS and FA oxidation offers less noxious therapy than conventional cisplatin treatment. Overall, this study highlights mitochondria as the metabolic Achilles heel of LUAD to develop new tailored medicine for the patients.

## Results

### The metabolic proteome of lung adenocarcinomas

**Fig. 1:**
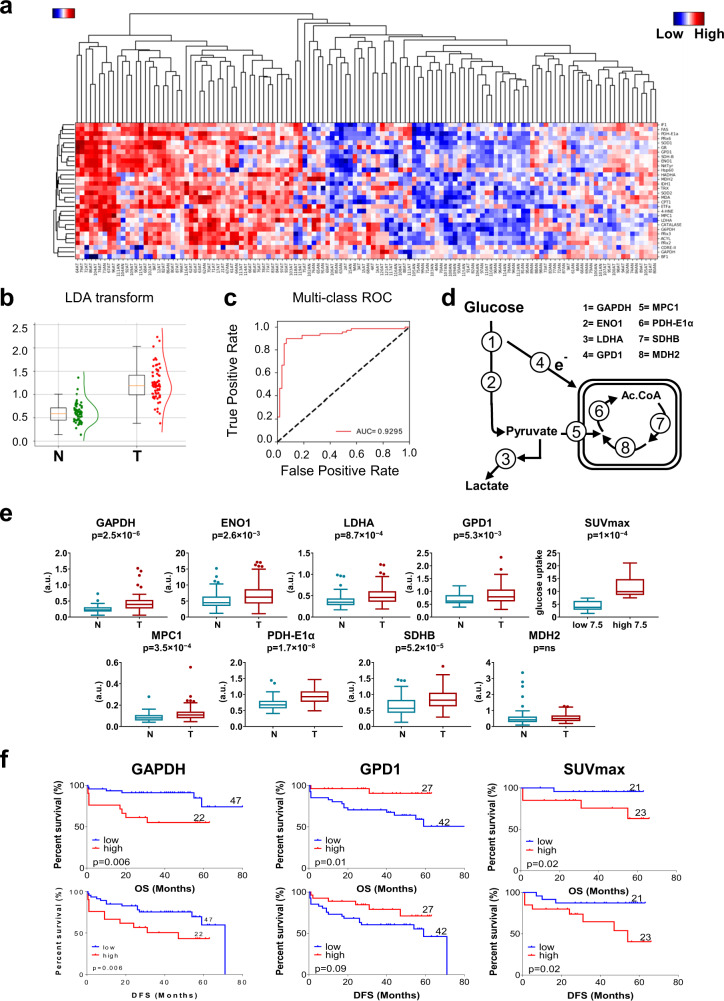
Analysis of protein biomarkers: Proteins of glycolysis and pyruvate oxidation. **a** Hierarchical clustering analyses of lung adenocarcinoma (T) and adjacent non-tumor (N) lung biopsies using the indicated proteins markers. Protein expression scores are shown normalized to a Ɲ (−0.5–0.5) and marked according to a color scale (top right panel): red, high; white, normal and blue, low expression. **b**, **c** Illustrate the distribution of N (green box/line) and T (red box/line) samples by Linear Discriminant Analysis (**b**), and the probability of detection by a Multiclass ROC curve (**c**) using CPT1, HADHA, FAS, Hsp60, SOD2 and TRX as combination of biomarkers. The Area Under the Curve (AUC) is indicated. **d** Representative scheme illustrating four steps of glycolysis catalyzed by glyceraldehyde 3-phosphate dehydrogenase (GAPDH), enolase 1 (ENO1), lactate dehydrogenase A (LDHA) and glycerol 3-phosphate dehydrogenase (GPD1) and four steps involved in the oxidation of pyruvate in mitochondria catalyzed by the pyruvate carrier (MPC1), pyruvate dehydrogenase E1 component subunit alpha (PDH-E1a), succinate dehydrogenase subunit B (SDHB) and malate dehydrogenase 2 (MDH2). **e** Expression. The Y axis indicates the values of intensity (a.u) calculated by interpolation in the linear plot of HCT116 cells (Supplemental Fig. S3). *Box plots* represent the lowest, lower quartile, median, upper quartile and highest observations of each protein (a.u.) in tumor (T, *n* = 69) and non-tumor (N, *n* = 59) samples quantified by RPPA. The glucose uptake values (SUVmax, *n* = 44) assessed by FDG-PET/CT scans in carcinomas are also shown. *p*-values by Student’s *t*-test are shown. **f** Kaplan–Meier survival analysis shows the association of the indicated markers with overall (OS) and disease-free (DFS) survival of LUAD patients stratified by high (red line) and low (blue line) protein levels. Kaplan-Meier estimates are also shown for SUVmax values of PET scans. The log-rank *p*-values are also indicated.

RPPA were developed with antibodies against twenty-seven metabolic enzymes and biomarkers of protein oxidation, to provide quantitative information of the changes in the steady-state levels of the proteins and of oxidative damage brought about by cancer in lung biopsies (Supplemental Table S1). The specificity of the antibodies used was validated by western blot against lysates of A549 lung cancer cells (Supplemental Fig. S1). Unsupervised hierarchical clustering of tumor and NAT by the expression level of the biomarkers investigated distinguished two main groups of samples (Fig. 1a), one enriched in tumors (85% of the samples; Fig. 1a, to the left), and the other one containing the majority of NAT (88% of NAT; Fig. 1a to the right). However, in this second group, there was a relevant number of tumor biopsies (35% of the tumors; Fig. 1a to the right). Linear Discriminant Analysis (LDA) data transformation and classifier found that the combination of five mitochondrial proteins (carnitine palmitoyltransferase 1, CPT1; hydroxyl-CoA dehydrogenase subunit α, HADHA; heat shock protein 60, HSP60; superoxide dismutase 2, SOD2 and thioredoxin, TRX) plus the cytoplasmatic fatty acid synthase (FAS), correctly classified 93% of the samples (Fig. 1b), allowing discrimination of tumor from NAT with high sensitivity and specificity as revealed in the Receiver Operating Characteristic (ROC) curve (Fig. 1c). Overall, these results highlight the profound changes in the proteome of metabolism in LUAD and support that mitochondrial proteins are primary targets in lung carcinogenesis.

Protein levels in the carcinomas were correlated with the clinico-pathological information and overall survival (OS) and disease-free survival (DFS) of the patients (Table 1), to identify potential metabolic biomarkers of lung cancer prognosis and tentative targets for therapy.

**Table 1:** Univariate and Multivariate Cox regression analysis of overall survival and disease-free survival in LUAD patients.

	Univariate Cox regression			
	Overall survival		Disease-free survival	
	HR (95% CI)	*p*-value	HR (95% CI)	*p*-value
**Size**				
<3 cm	1 (Reference)		1 (Reference)	
≥3 cm	3.3(0.94–12)	0.062	2.1(0.87–5)	0.102
**PET**				
<7.5	1 (Reference)		1 (Reference)	
≥7.5	9(1–77)	0.046	5.1(1.4–20)	0.016
**Stage**				
I	1 (Reference)		1 (Reference)	
II-III-IV	1.3(0.43–3.6)	0.681	1.1(0.48–2.6)	0.786
**GAPDH**				
Low	1 (Reference)		1 (Reference)	
High	4.2 (1.5–12)	0.005	2.5(1.1–5.5)	0.023
**GPD1**				
Low	1 (Reference)		1 (Reference)	
High	0.21(0.047–0.91)	0.037	0.44(0.18–1.1)	0.081
**CPT1**				
Low	1 (Reference)		1 (Reference)	
High	0.18(0.041–0.079)	0.023	0.4(0.16–1)	0.052
**ETFA**				
Low	1 (Reference)	0.016	1 (Reference)	0.009
High	0.16(0.036–0.71)		0.27(0.1–0.72)	
**HADHA**				
Low	1 (Reference)	0.025	1 (Reference)	0.023
High	0.18(0.042–0.081)		0.32(0.12–0.86)	
**CPT1/ACYL**				
Low	1 (Reference)		1 (Reference)	
High	0.11(0.026–0.5)	0.004	0.31(0.13–0.73)	0.008
**Hsp60**				
Low	1 (Reference)		1 (Reference)	
High	0.1(0.024–0.46)	0.003	0.33(0.14–0.78)	0.011
**βF1**				
Low	1 (Reference)		1 (Reference)	
High	8.3(1.9–37)	0.005	2.6(1.1–6)	0.029
**IF1**				
Low	1 (Reference)		1 (Reference)	
High	0.2(0.07–0.57)	0.003	0.3(0.13–0.74)	0.009
**IF1/ βF1**				
Low	1 (Reference)		1 (Reference)	
High	0.16(0.06–0.44)	0.001	0.23(0.1–0.51)	0.001
**PRX6**				
Low	1 (Reference)		1 (Reference)	
High	0.11(0.032–0.4)	0.001	0.34(0.15–0.76)	0.008
**SOD2**				
Low	1 (Reference)		1 (Reference)	
High	4.3(1.6–12)	0.004	2.8(1.2–6.5)	0.016
**PRX3**				
Low	1 (Reference)		1 (Reference)	
High	3.3(1.1–10)	0.041	2.5(1.1–5.7)	0.037
**PRX3/PRX6**				
Low	1 (Reference)		1 (Reference)	
High	21(2.8–16)	0.003	3.7(1.5–8.9)	0.004

	Multivariate Cox regression				
	Overall survival			Disease-free survival	
	HR (95% CI)	*p*-value		HR (95% CI)	*p*-value
**GAPDH**			**GAPDH**		
Low	1 (Reference)		Low	1 (Reference)	
High	7.167(1.845–27.84)	0.004	High	3.12(1.152–8.46)	0.025
**CPT1**					
Low	1 (Reference)			**-**	
High	0.082(0.013–0.53)	0.008			
			**ETFa**		
	-		Low	1 (Reference)	
			High	0.13(0.037–0.47)	0.002
			**HADHA**		
	-		Low	1 (Reference)	
			High	0.25(0.076–0.79)	0.019
**BF1**					
Low	1 (Reference)			**-**	
High	4.524(0.955–21.43)	0.057			
			**IF1**		
			Low	1 (Reference)	
	-		High	0.11(0.025–0.51)	0.005
			**Hsp60**		
	-		Low	1 (Reference)	
			High	4.56(1.173–17.71)	0.028
**SOD2**			**SOD2**		
Low	1 (Reference)		Low	1 (Reference)	
High	8.314(2.112–32.74)	0.002	High	13.29(3.338–52.90)	0.001

### The signature of glycolysis and pyruvate oxidation

The enzymes of glycolysis glyceraldehyde-3-phosphate dehydrogenase (GAPDH), enolase 1 (ENO1) and lactate dehydrogenase (LDHA) (Fig. 1d) and glycerol-3-phosphate dehydrogenase (GPD1) which shuttles electrons to mitochondria (Fig. 1d), revealed a significant increase in carcinomas when compared to NAT (Fig. 1e). The higher content of GAPDH negatively correlated with OS and DFS of the patients in Kaplan-Meier estimates (Fig. 1f), a finding that was also confirmed by univariate Cox regression analysis (Table 1). Multivariate Cox models indicated that GAPDH is also an independent predictor of OS and DFS of the patients (Table 1), supporting that an enhanced glycolytic flux compromises patients’ prognosis. Estimation of the glucose uptake by the SUVmax of ^18^FDG in PET scans of the patients (Fig. 1e) indicated that an enhanced glucose uptake is a functional biomarker of bad OS and DFS (Fig. 1f, Table 1), in agreement with previous findings [28]. In contrasts, increased tumor levels of GPD1 (Fig. 1e) positively correlated with better OS and DFS of the patients (Fig. 1f and Table 1), suggesting that preservation of the shuttling of electrons to mitochondria could favor patients’ prognosis.

With the exception of the TCA cycle enzyme malate dehydrogenase (MDH2) (Fig. 1e), protein levels of the mitochondrial pyruvate carrier (MPC1), and pyruvate dehydrogenase E1 alpha subunit (PDHE1α) and succinate dehydrogenase B (SDHB) of the TCA cycle (Fig. 1d), increased in LUAD when compared to NAT (Fig. 1e). None of these proteins showed significant correlations with patients’ survival.

### The signature of FA metabolism

**Fig. 2:**
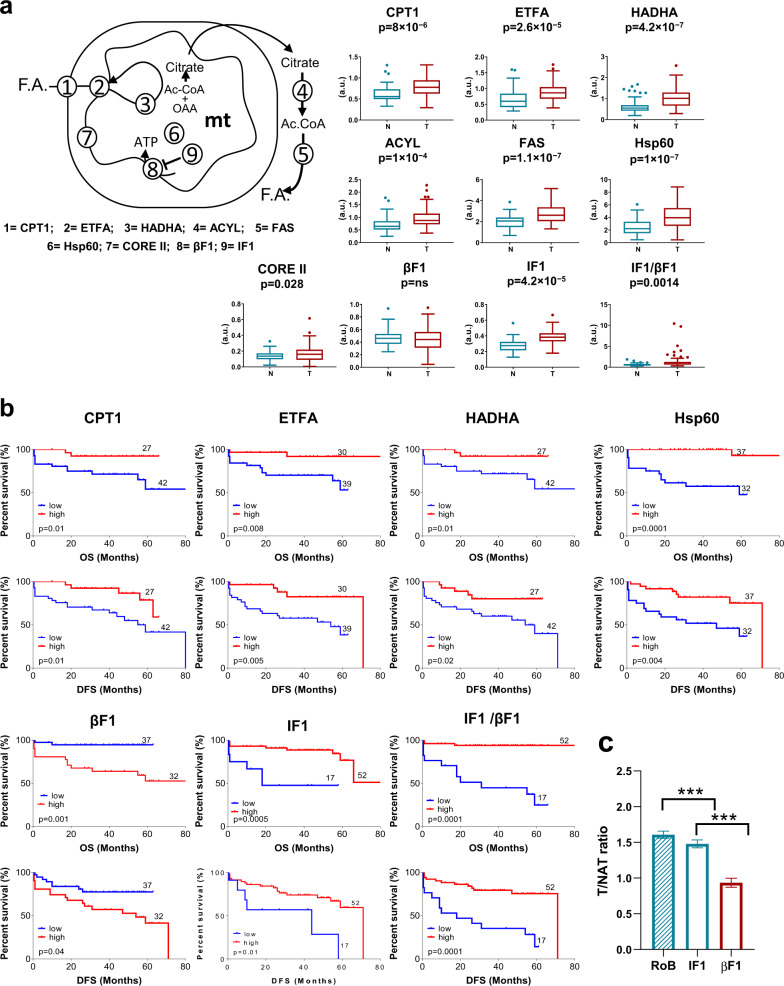
Oxidation and synthesis of FA and OXPHOS in lung cancer: Expression and prognosis. **a** Representative scheme illustrating three steps of fatty acid oxidation catalyzed by carnitine palmitoyltransferase 1 (CPT1), electron transfer flavoprotein subunit alpha (ETFA) and hydroxyacyl-CoA dehydrogenase subunit alpha (HADHA), two steps of the cytoplasmic synthesis of FA catalyzed by ATP citrate lyase (ACLY) and fatty acid synthase (FAS) and three steps of the OXPHOS system that involved ubiquinol-cytochrome c reductase core protein 2 (CORE II) of complex III, the β catalytic subunit (βF1) of the ATP synthase and its inhibitory protein IF1. The structural mitochondrial heat shock protein 60 (Hsp60) is also depicted. The Y-axis indicates the values of intensity (a.u) calculated by interpolation in the linear plot of HCT116 cells (Supplemental Fig. S3). *Box plots* show protein levels (a.u.) of each marker and of the IF1/βF1 ratio in tumor (T, *n* = 69) and non-tumor (N, *n* = 59) samples quantified by RPPA. *p*-values by Student’s *t* test are shown. **b** Kaplan–Meier survival analysis shows the association of the indicated markers with overall (OS) and disease-free (DFS) survival of LUAD patients stratified by high (red line) and low (blue line) protein levels or value of the IF1/βF1 ratio. The log-rank *p*-values are also indicated. **c** The T/NAT ratio of IF1 and β-F1-ATPase (βF1) in adenocarcinomas. The mean T/NAT ratio of the Rest of Biomarkers (RoB) studied is shown for comparison. ***p < 0.0001 by ANOVA with Tukey’s multiple comparison test.

Steady-state levels of CPT1, the electron transfer flavoprotein subunit α (ETFA) and HADHA, three proteins involved in the mitochondrial transport and oxidation of FA, increased significantly in tumor biopsies (Fig. 2a). High levels of the three proteins in carcinomas predicted better OS and DFS for lung cancer patients (Fig. 2b, Table 1). In multivariate Cox models CPT1, and ETFA and HADHA, were found as independent predictors of OS and DFS, respectively (Table 1).

The content of both ATP citrate lyase (ACYL) and fatty acid synthase (FAS), which are involved in the synthesis of FA, increased significantly in tumor biopsies when compared to NAT (Fig. 2a). However, none of these enzymes showed correlation with OS or DFS of the patients.

### The signature of OXPHOS

Except for the protein content of β-F1-ATPase of OXPHOS (Fig. 2a), the amount of ubiquinol-cytochrome c reductase core protein 2 (COREII) of complex III of the respiratory chain, HSP60 and of ATPase inhibitory factor 1 (IF1) increased significantly in tumor when compared to NAT (Fig. 2a). Consistent, with the lack of changes in β-F1-ATPase expression in adenocarcinomas its T/NAT ratio remained unaltered whereas the T/NAT ratios of IF1, or of any of the biomarkers already mentioned (RoB), sharply increased (Fig. 2c). Interestingly, Kaplan-Meier (Fig. 2b) and Cox regression analysis (Table 1) indicated that a high tumor content of β-F1-ATPase predicted bad OS and DFS for the patients (Fig. 2b, and Table 1). Moreover, β-F1-ATPase content is an independent predictor of bad prognosis (Table 1). In contrast, a high tumor content of HSP60 and IF1 predicted better OS and DFS (Fig. 2b, and Table 1), being both IF1 and HSP60 independent predictors of recurrence of the disease as assessed in multivariate Cox models (Table 1). The ratio between IF1 and its target on the ATP synthase, β-F1-ATPase (Fig. 2a), is higher in carcinomas and predicted better OS and DFS for LUAD patients (1.69 ± 0.30 (*n* = 52) versus 0.50 ± 0.02 (*n* = 17), *p* < 0.01 for good or bad prognosis groups, respectively) (Fig. 2b; Table 1).

### The antioxidant signature

**Fig. 3:**
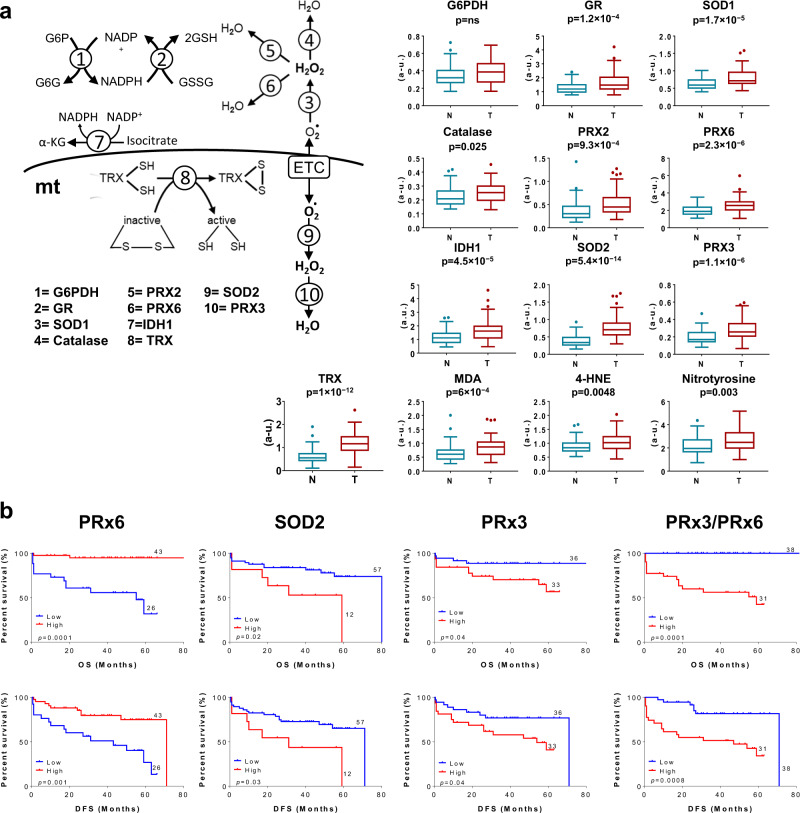
Antioxidant response and oxidative stress in lung cancer: Expression and prognosis. **a** Representative scheme illustrating ten relevant steps involved in the antioxidant response including cytoplasmic glucose-6-phosphate dehydrogenase (G6PDH), glutathione reductase (GR), superoxide dismutase 1 (SOD1), catalase, peroxiredoxin 2 (PRX2), peroxiredoxin 6 (PRX6) and isocitrate dehydrogenase 1 (IDH1), and mitochondrial thioredoxin reductase (TRX), superoxide dismutase 2 (SOD2) and peroxiredoxin 3 (PRX3) enzymes. *Box plots* show the expression level of each protein, as well as the content of malondialdehyde (MDA), 4-hydroxynonenal (4-HNE) and nitrotyrosine protein adducts in non-tumor (N, *n* = 59) and tumor (T, *n* = 69) samples quantified by RPPA. The Y-axis indicates the values of intensity (a.u) calculated by interpolation in the linear plot of HCT116 cells (Supplemental Fig. S3). *p*-values by Student’s *t*-test are shown. **b** Kaplan–Meier survival analysis shows the association of the indicated markers with overall (OS) and disease-free (DFS) survival of LUAD patients stratified by high (red line) and low (blue line) protein levels or value of the PRX3/PRX6 ratio. The log-rank *p*-values are also indicated.

The tumor content of the proteins involved in the antioxidant response glucose-6-phosphate dehydrogenase (G6PDH), glutathione reductase (GR), superoxide dismutase 1 and 2 (SOD1 and SOD2), catalase, peroxiredoxin 2, 3 and 6 (PRX2, PRX3, PRX6), NADP^+^-dependent isocitrate dehydrogenase 1 (IDH1) and thioredoxin (TRX) were significantly augmented when compared to its content in NAT (Fig. 3a). Consistently, protein oxidative damage, as assessed by malondialdehyde (MDA), 4-hydroxynonenal (4-HNE) and nitrotyrosine modification of proteins in tissue extracts was also increased in tumors (Fig. 3a). Remarkably, a high tumor content of cytoplasmic peroxiredoxin 6 (PRX6) predicted better OS and DFS for lung cancer patients both by Kaplan-Meier estimates (Fig. 3b) and in univariate Cox regression analysis (Table 1), suggesting that the activation of the cytoplasmic antioxidant response protects from cancer progression. In contrasts, a high tumor content of the mitochondrial enzymes involved in scavenging superoxide radical (SOD2) and hydrogen peroxide (PRX3), predicted poor OS and DFS for the patients (Fig. 3b, Table 1). In multivariate Cox models SOD2 was an independent predictor of OS and DFS of the patients (Table 1). Combination of the cytoplasmic and mitochondrial antioxidant responses, using the PRX3/PRX6 ratio, also predicted worse OS and DFS for the patients when the value of the ratio was high (0.18 ± 0.01 (*n* = 31) versus 0.08 ± 0.01 (*n* = 38), *p* < 3.4E-11 for bad and good prognosis groups, respectively) (Fig. 3, Table 1), supporting that mitochondrial oxidative stress negatively affects prognosis.

### The integration of metabolic and redox proteins in lung cancer

Changes in the tissue content of the proteins allowed the establishment of linear correlations to infer potential mechanisms underpinning the metabolic reprograming in LUAD. Supplemental Fig. S3 (upper left panel) shows a matrix of the statistical significance of the linear correlations that exist between the content of metabolic and redox proteins in tumor (T), NAT (N) and T + N (All) samples (see insets in panels of Supplemental Fig. S3). In summary, significant correlations were obtained for the changes in protein content between enzymes that belong to the same and/or to different metabolic pathways in tumors (T), NAT (N) and tumors+NAT (T + N). It is remarkable that these correlations were not altered when compared tumor to NAT, being their significance augmented when considering All samples (Supplemental Fig. S3), suggesting a concerted co-regulation of the pathways involved. Interestingly, the content of several of the enzymes of metabolism correlated with those involved in scavenging hydrogen peroxide, superoxide and hydroperoxides (Supplemental Fig. S3). Moreover, several of the enzymes of glycolysis, TCA cycle and FA oxidation also correlated with overall oxidative damage of proteins (Supplemental Fig. S3), supporting a link between the increase metabolic activity, the generation of reactive oxygen species (ROS), oxidative damage and the response of the antioxidant system. Altogether, the results suggest that the mechanism underlying the co-regulation of the pathways might stem from the different flux of ROS produced during metabolic activity. In fact, ROS have been recently shown to play a role in regulating the turnover of the cellular proteome during metabolic reprograming [29].

### A signature of LUAD metastasis

**Fig. 4:**
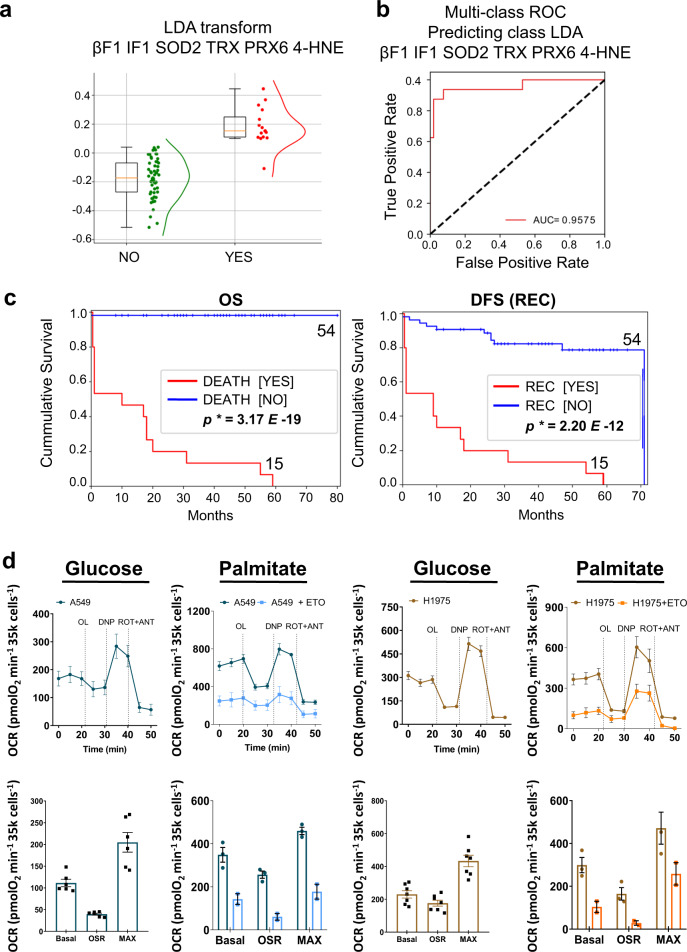
A proteomic signature of lung cancer prognosis. LDA classifier found βF1 - IF1 - SOD2 - TRX - PRX6–4-HNE as the best combination of markers of OXPHOS (βF1; IF1), antioxidant response (SOD2; TRX; PRX6) and oxidative damage (4-HNE) able to predict patients’ fate in 69 lung adenocarcinomas. **a** *Box plots* show the distribution of the patients who did not die (NO, green dots/line; *n* = 54) and patients who died (YES, red dots/line; *n* = 15) calculated by Linear Discriminant Analysis (LDA) data transformation of the combination of markers mentioned. **b** Multi-class ROC curve indicating the performance measurement for the two-class classification model (NO, non-dead; YES, dead people) at various thresholds settings. The Area Under the Curve (AUC) is indicated. **c** Kaplan–Meier curves for overall (OS) and disease-free (DFS) survival probability for the cohort of 69 lung adenocarcinoma patients stratified by non-dead [NO] (blue line) and dead [YES] (red line) people using the combination of markers mentioned. The log-rank *p*-values are indicated. **d** Respiratory profile of A549 and H1975 lung adenocarcinoma cells using glucose (*n* = 6) or palmitate (*n* = 3) as main respiratory substrates. The respiratory profiles of cells pretreated (15 min) with the CPT1 inhibitor etomoxir (400 µM) (light blue and orange traces) are also shown. The histograms show the quantification of basal-, oligomycin sensitive- (OSR) and maximum- (MAX) respiration of the cells treated or not with etomoxir. OCR Oxygen consumption rate, OL Oligomycin, DNP 2,4-dinitrophenol, ROT Rotenone; ANT Antimycin A. Bars are the mean ± SEM of the indicated (*n*) biological replicates.

LDA classifier invariably found that more than 50% of the minimal combinations of biomarkers that are able to predict death or recurrence of the disease with high specificity and sensitivity are markers of the antioxidant signature in combination with those involved in OXPHOS, emphasizing the relevance of these pathways as hallmark of LUAD progression (Fig. 4a–c). In fact, the “proteomic signature of prognosis/metastasis” results from the combination of β-F1, IF1, SOD2, TRX, PRX6 and 4-HNE, which contains four biomarkers of the oxidative stress response and the two proteins that directly regulate ATP synthesis in mitochondria. The prognosis signature correctly classified 96% of the patients (Fig. 4a, b) with high specificity and sensitivity (Fig. 4b) and high power to predict survival or recurrence of the disease in LUAD patients (Fig. 4c).

### Overexpression of IF1 prevents metastatic disease

**Fig. 5:**
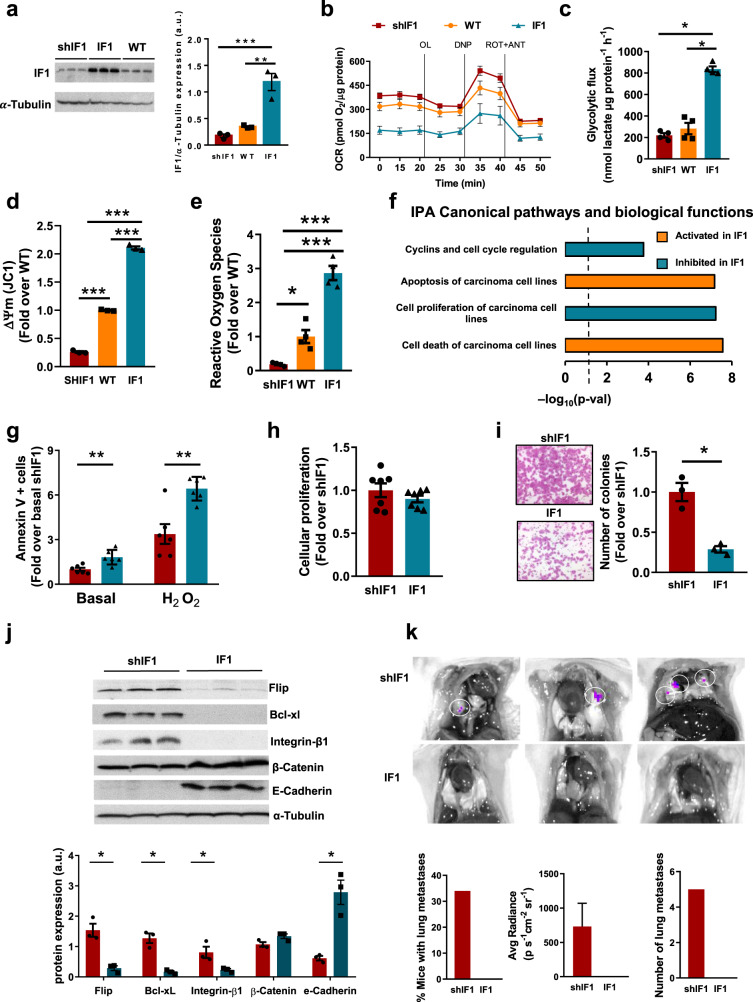
Overexpression of IF1 prevents metastatic disease. IF1 was overexpressed (IF1, blue bar) or silenced (shIF1, red bars) in parental (WT, orange bars) A549 cells. **a** Quantification of IF1 expression. Tubulin is used as loading control (*n* = 3). Respiratory profile (n = 6) (**b**) and glycolytic flux (*n* = 4) (**c**) of the cells. **d** Mitochondrial membrane potential (ΔΨm) using JC1 as probe and expressed as fold change of WT (*n* = 3). **e** ROS production using H_2_DCFDA fluorescence expressed as fold change of WT (*n* = 4). **f** IPA canonical pathways and biological functions activated (orange bars) or inhibited (blue bars) in IF1 overexpressing cells when compared to shIF1 cells. Z-score indicates the overall predicted activation/inhibition state (*n* = 3). **g** Cell death of A549 cells in the absence (basal) or presence of 60 µM hydrogen peroxide (H_2_O_2_) expressed as fold change of shIF1 cells under basal conditions (*n* = 6). **h** Cellular proliferation (*n* = 7). **i** Representative images of the Matrigel invasion assays of shIF1 (upper) and IF1 (lower) cells at 72 h (*n* = 3). The histogram shows invasion normalized to shIF1 cells. **j** Expression of *anoikis*, apoptosis, extracellular matrix and epithelial–mesenchymal transition proteins in three different replicates of shIF1 and IF1 cells. Tubulin is used as loading control (*n* = 3). **k** Bioluminescence imaging of metastasis developed in lung of mice injected with shIF1 (upper panels) and IF1 (lower panels) A549 cells. The histograms show the percentage of mice with metastases, bioluminescence intensity and number of metastases. Results shown are means ± S.E.M; *p ≤ 0.05; **p ≤ 0.01 and ***p ≤ 0.001 by One-way ANOVA and Tukey’s multiple comparisons tests (**a**, **c**, **d**, **e**) and Student’s *t*-test (**g**–**j**).

The overexpression of IF1 in NSCLC as assessed by non-quantitative immunohistochemistry has been suggested to predict bad patients’ prognosis [30] in contrast with our findings (Fig. 2b; Table 1). Hence, we next investigated the molecular basis of the anti-oncogenic role of IF1 in LUAD cells by developing stable A549 cells overexpressing (IF1) or silencing IF1 (shIF1), and PC9 IF1-knockout (KO-IF1) cells. The expression of IF1 in the resulting cells was assessed by immunoblotting and compared to the parental lines (Fig. 5a and Supplementary Fig. S2a). In agreement with previous results in other cancer cells [31] and in neurons of IF1-genetically modified mice [32], cells expressing high levels of IF1 showed significant inhibition of mitochondrial respiration (Fig. 5b and Supplementary Fig. S2b) and an enhanced glycolysis (Fig. 5c and Supplementary Fig. S4c). In contrasts, shIF1 or KO-IF1 cells showed increased respiration (Fig. 5b and Supplementary Fig. S2b) and lower rates of glycolysis (Fig. 5c and Supplementary Fig. S2c) when compared to parental cells. Likewise, and in agreement with recent findings [32], the mitochondrial membrane potential (Fig. 5d and Supplementary Fig. S2d) and the production of reactive oxygen species (ROS) (Fig. 5e) were significantly augmented with IF1 dose.

Ingenuity Pathway Analysis (IPA) (Fig. 5f) of a targeted transcriptomic study in A549 cells (Supplementary Table S2) suggested that the overexpression of IF1 promotes apoptosis and cell death of carcinoma cells (Fig. 5f). Consistently, shIF1 cells were more resistant to death or hydrogen peroxide-induced cell death than IF1 cells (Fig. 5g). Despite IPA analysis suggested that cyclin and cell cycle regulation and the proliferation of cancer cells were downregulated in IF1 cells (Fig. 5f), the rates of cellular proliferation showed no relevant differences between shIF1 and IF1 cells (Fig. 5h). Cellular invasion assays indicated that both IF1-silenced A549 (Fig. 5i) or KO-IF1 PC9 (Supplementary Fig. S2f) cells have higher invasive capacity than IF1 expressing cells. Consistently, shIF1 A549 (Fig. 5j) and KO-IF1 PC9 (Supplementary Fig. S2g) cells showed enhanced expression of cFLIP (FLICE-inhibitory protein, a potent inhibitor of cell death upon cellular detachment), of the anti-apoptotic Bcl-xL and of the pro-metastatic β1-integrin when compared to IF1 expressing cells. In contrast, IF1 expressing cells showed the overexpression of E-cadherin (Fig. 5j and Supplementary Fig. S2g), a glycoprotein involved in cell adhesion. These results also support that LUAD cells with a low content of IF1 have greater potential for tumor metastasis, confirming the transcriptomic results (Supplementary Table S2). To assess experimental lung metastatic disease in vivo [33], rather than using the spontaneous metastasis assay [34], we intravenously injected shIF1 and IF1 A549 cells into nude mice. Congruently, only shIF1 cells developed into lung metastases (Fig. 5k). Overall, the results suggest that overexpression of IF1 in LUAD cells, despite favoring a glycolytic phenotype, promotes a less invasive phenotype by repressing OXPHOS and thus underpinning the lower metastasis and better prognosis of LUAD patients bearing tumors with high levels of IF1.

### Adenocarcinomas efficiently oxidized FA

Mitochondrial electron transport chain is necessary for tumor growth [35]. Moreover, the metabolic proteome of LUAD revealed that two mitochondrial enzymes involved in the oxidation of FA, CPT1 and HADHA, are relevant biomarkers of the disease (Fig. 1b, c and Table 1). Hence, we investigated the mitochondrial respiratory capacity of glucose and palmitate in A549 and H1975 lung adenocarcinoma cells. The underlying idea was to find out the relevance of FA oxidation as energy source in LUAD (Fig. 4d) and its potential for a targeted therapy. Both cell lines efficiently oxidized palmitate (Fig. 4d). Etomoxir, an inhibitor of CPT1, blocked the oxidation of palmitate (Fig. 4d), supporting its use to target mitochondrial FA oxidation as suggested previously [36], even though it has off-target effects on other mitochondrial proteins involved in OXPHOS [37], limiting its target specificity.

### Blocking the assimilation/oxidation of FA arrests tumor growth

A preliminary study was developed to assess the potential of targeting FA assimilation/oxidation for the treatment of LUAD in vivo (Supplementary Fig. S4). To this aim, nude mice were implanted with A549-Luc cells in the rear flanks and after ~ 30 days the animals were allotted into four different groups for treatment initiation. Mice were treated or not (control group, CRL) with a daily i.p. injection, five days a week, of 240 mg kg^−1^ of orlistat (ORLI), an inhibitor of gastrointestinal lipases to prevent the assimilation of FA from the diet, or 50 mg kg^−1^ of etomoxir (ETO), to prevent the oxidation of FA in mitochondria. A fourth group was administered the β1-adrenergic blocker nebivolol (NEB, 10 mg kg^−1^), which we recently showed inhibits OXPHOS in different cancer cells including lung A549 cells [18]. Moreover, nebivolol effectively arrests the in vivo growth of colon and breast carcinomas [18] and squamous cell carcinomas [38]. Mechanistically, the inhibition of the β1-adrenergic cascade in cancer cells leads to the upregulation of the mitochondrial content of ATPase inhibitory factor 1 (IF1) and also prevents the phosphorylation of Complex I subunits such as NDUFS7 [18]. Therefore, the effect of nebivolol in mitochondria is double, it inhibits both the ATP synthase and Complex I activities; in other words, it arrests OXPHOS. Moreover, the inhibition of the β1-adrenergic cascade also limits glycolysis of endothelial cells causing cell cycle arrest [18]. Altogether, nebivolol promotes a metabolic and redox crisis that restricts the growth of carcinomas [18]. Interestingly, the three treatments revealed significant inhibition of tumor growth after 30 days of treatment when compared to non-treated mice (Supplementary Fig. S4), supporting the idea that interfering the assimilation of FA or its mitochondrial oxidation could represent additional strategies for LUAD treatment.

**Fig. 6:**
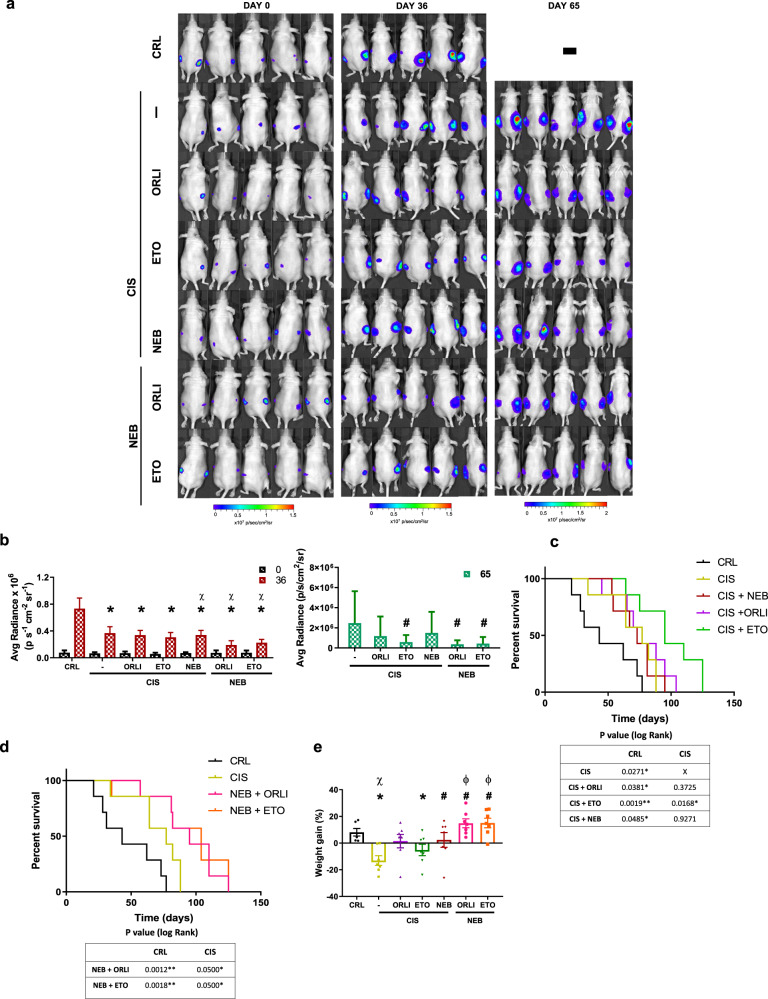
Inhibition of fatty acid oxidation and OXPHOS provide an effective and better tolerated therapeutic strategy against LUAD. A549-Luc cells were injected into the flanks of nude mice. When tumors reached a volume of ~100 mm^3^, mice were treated with saline (CRL; black trace and bars; *n* = 7), 5 mg kg^−1^ cisplatin (CIS, yellow trace and bar; *n* = 7); 5 mg kg^−1^ cisplatin plus 240 mg kg^−1^ orlistat (CIS + ORLI, purple trace and bar; *n* = 7); 5 mg kg^−1^ cisplatin plus 40 mg kg^−1^ etomoxir (CIS + ETO; green trace; *n* = 7), 5 mg kg^−1^ cisplatin plus 10 mg kg^−1^ nebivolol (CIS + NEB, red trace and bars; *n* = 7), 10 mg kg^−1^ nebivolol plus 240 mg kg^−1^ orlistat (NEB + ORLI, pink trace and bars; *n* = 7) or 10 mg kg^−1^ nebivolol plus 40 mg kg^−1^ etomoxir (NEB + ETO, orange trace and bars; *n* = 7). Mice were euthanized when tumor volume reached 1500 mm^3^. **a** Upper panels show representative images of the bioluminescence of A549-Luc cells in mice after 0, 36, and 65 days of initiation of the treatments. **b)** Histograms show the quantification of light emission of the cells (CRL, *n* = 14; CIS, *n* = 14; CIS + ORLI, *n* = 14; CIS + ETO, *n* = 14; CIS + NEB *n* = 14; NEB + ORLI, *n* = 14; NEB + ETO, *n* = 14) at days 36 (left) and 65 (right) days of treatment. **c, d** Kaplan–Meier survival analysis. The log-rank test *p*-value is shown. **e** Percentage weight gain of mice in response to treatments. *p < 0.05 when compared to CRL and ^#^*p* < 0.05 when compared to CIS by two-sided Student’s *t*-test; χ and ^ϕ^*p* < 0.05 when compared to CRL and CIS by one-way ANOVA and Dunnett’s multiple comparisons tests, respectively. Bars indicate the mean ± SEM of the indicated number of tumors or mice.

A more ambitious study was designed to compare the in vivo efficacy of cisplatin alone or in combination with orlistat, etomoxir or nebivolol for the treatment of lung cancer (Fig. 6a, b). In addition, we studied the combinations of the metabolic inhibitors orlistat+nebivolol or etomoxir+nebivolol, as alternatives to conventional cisplatin chemotherapy (Fig. 6a, b). Cisplatin alone, or in any combination tested, significantly arrested tumor growth (Fig. 6a, b) and extended lifespan when compared to non-treated mice (Fig. 6c). Only the combination of cisplatin+etomoxir significantly delayed tumor growth and extended lifespan of mice when compared to mice treated with cisplatin (Fig. 6b, c), suggesting that the inhibition of FA oxidation could provide a complementary target for the treatment of LUAD. In general, and when compared to non-treated mice, tumor reduction in cisplatin-treatments resulted from a diminished proliferation (Ki67+ cells in Supplementary Fig. S5a, b) and an enhanced cell death (c-casp3+ cells in Supplementary Fig. S5a, c) in the carcinomas. Moreover, cisplatin treatments significantly reduced tumor angiogenesis (SMA staining in Supplementary Fig. S5a, d), what resulted in a significant increase in the necrotic area of the carcinomas (Supplementary Fig. S5a, e).

Interestingly, the combinations of nebivolol+orlistat or nebivolol+etomoxir also delayed tumor growth (Fig. 6a, b) and extended lifespan of mice when compared to cisplatin treatment (Fig. 6d). It is worth noting that blocking β1-adrenergic signaling in combination with limiting the assimilation/oxidation of FA had no negative effect on welfare and body weight gain of mice whereas cisplatin treatments did (Fig. 6e). Tumor reduction with the combined nebivolol treatments also resulted from a diminished proliferation (Supplementary Fig. S5a, b) and an enhanced cell death (Supplementary Fig. S5a, c) in the carcinomas, as it was the case in cisplatin treatments. Likewise, nebivolol treatments also significantly reduced tumor angiogenesis (Supplementary Fig. S5a, d) resulting in a significant increase in the necrotic area of the carcinomas (Supplementary Fig. S5a, e). Overall, these findings support that preventing the assimilation/oxidation of FA in combination with the inhibitory actions of the β1-adrenergic blocker in OXPHOS and angiogenesis [18] have potential for the treatment of LUAD patients.

## Discussion

Genetic studies have provided a wealth of information regarding the molecular alterations in oncogenes and ICIs that act as genetic drivers of LUAD [2, 3]. However, despite the success of some targeted therapies, resistance inevitably occurs [2, 3]. More recently, integrative proteomic [10] and proteogenomic [12] characterization of LUAD and paired NAT, have revealed novel candidate metabolic pathways to support that cancer patient may benefit from therapies targeting the metabolic dependencies of their carcinomas, either as single therapeutic agents or in combination with other chemo- or immunotherapies [15–18]. However, to advance in this field, it is required the implementation of comprehensive quantitative approaches to determine the content and relative relevance of the enzymes that steer the pathways of tumor metabolism. In this regard, protein expression profiling by high-throughput RPPA offers a powerful quantitative immunological approach for detailed characterization of the metabolic proteome of cancer and for the development of therapeutic strategies [26, 27]. A major limitation of RPPA technology is the availability of validated antibodies that should be monospecific recognizing only the protein of interest [39], as shown in this study.

Herein, we have followed a comparative protein expression profiling of tumor and NAT in a cohort of LUAD by RPPA using specific antibodies to portray a relevant fraction of the metabolic reprogramming experienced by this type of carcinomas. NAT is molecularly different from normal tissue [40]. However, when carcinomas are compared to NAT, they show significant increases in the steady-state level of the majority of the enzymes studied, complementing the findings of a recent proteomic characterization of LUAD [10]. Changes in the protein content of six enzymes discriminated tumor from non-tumor biopsies. Interestingly, five out of six of these enzymes are resident in mitochondria, strongly supporting that the mitochondrial proteome is a most relevant target in metabolic reprogramming of LUAD. Moreover, several of these proteins are independent predictors of survival and/or of disease recurrence. Interestingly, the comparison of the prognostic significance of RPPA biomarkers by their absolute expression level, or by Z-score normalized expression, reveal that they have higher sensitivity than those derived from RNA expression analysis (Supplementary Tables S5). Congruently, proteomic data derived from different immunological studies support that the absolute expression level of these proteins correlate with survival of the patients in LUAD and other cancer studies (Supplementary Table S5) (see [24] for review). Hence, differences in LUAD prognosis using RNA or protein as biomarkers might stem from the post-transcriptional mechanisms that regulate proteins’ expression which affect a large number of mitochondrial proteins [41].

**Fig. 7:**
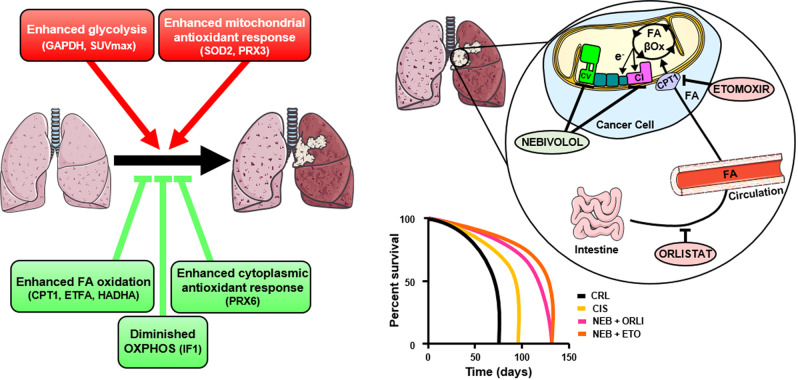
Graphical abstract and highlights. The metabolic profile of LUAD provides biomarkers of prognosis and for targeted therapies. The schematic summarizes the main metabolic signatures involved in LUAD progression. On the top (in red), an enhanced glucose uptake (SUVmax), glycolysis (GAPDH) and mitochondrial antioxidant response (SOD2, PRX3) are markers of bad prognosis for the patients. Below (in green), an enhanced fatty acid oxidation (CPT1, ETFA, HADHA) and cytoplasmic antioxidant response (PRX6) are markers of good prognosis. Likewise, a reduced OXPHOS (IF1) also predicts good prognosis for LUAD patients. The representation highlights the prominent role of FA β-oxidation and mitochondrial OXPHOS as targets for effective and less noxious therapy of LUAD. Orlistat (pink) inhibits gastrointestinal lipases restraining the uptake of FA into the circulation. Etomoxir (pink) inhibits CPT1 preventing the import of FA into mitochondria. Both orlistat and etomoxir prevent β-oxidation of FA and the supply of electrons to the respiratory chain. Nebivolol (light green) is a β1-adrenergic blocker that inhibits OXPHOS at complexes I (red) and V (ATP synthase) (green) generating a metabolic and redox crisis in cancer cells. The combination of metabolic inhibitors targeting mitochondrial activities are better tolerated and significantly increase lifespan of mice bearing LUAD when compared to cisplatin treatment.

Our data stressed that the upregulation of glucose uptake (SUVmax) and glycolysis (GAPDH) compromise patients’ survival in agreement with previous reports [28] and recent GSEA data of the LUAD proteome [10] (Fig. 7). Likewise, our results highlight that the upregulation of the mitochondrial antioxidant response (SOD2, PRX3) also compromise the survival of LUAD patients (Fig. 7). On the contrary, the upregulation of enzymes that feed carbon skeletons for mitochondrial oxidation (CPT1, HADHA, ETFA), mitochondrial structure (HSP60) or the cytoplasmic antioxidant response (PRX6) are favorable biomarkers of LUAD prognosis (Fig. 7). Similarly, GSEA of the proteomic data of LUAD also stressed that enrichment of FA oxidation indicate good prognosis for LUAD patients [10]. Overall, the comprehensive and quantitative account of the changes in metabolic enzymes by RPPA provides additional insights into the molecular features of LUAD that could help to define new anticancer strategies by targeting specific metabolic differences.

Special consideration deserves the two proteins that control OXPHOS that are included in the prognosis/metastatic signature (β-F1-ATPase and IF1). Despite the large increase in the structural HSP60 and of other mitochondrial proteins, the content of β-F1-ATPase, which is the catalytic subunit of the ATP synthase and bottleneck in OXPHOS, showed no relevant changes in LUAD. In fact, the “bioenergetic signature” [42] of the carcinomas was significantly diminished when compared to NAT (0.48 ± 0.06 *versus* 1.08 ± 0.08, *P* < 0.001 for tumor and NAT, respectively), in agreement with previous similar findings in lung cancer [28, 43], suggesting that the capacity for the synthesis of ATP by OXPHOS is somehow limited in mitochondria of LUAD when compared to NAT mitochondria. Regardless of the absence of changes in β-F1-ATPase, it should be noted, in agreement with the recent identification of two (low and high) bioenergetic subtypes of LUAD [23], that the subset of patients with a high content of β-F1-ATPase in their tumors have worse prognosis. Similar findings have been obtained in large cohorts of breast cancer [44] and melanoma patients [45], supporting that the bioenergetic activity of mitochondria, despite the above mentioned relative “limitation” of OXPHOS, is required for metastatic disease and cancer progression [19, 35, 46].

It should be noted that in addition to the content of β-F1-ATPase, the mitochondrial ATP synthase is also regulated by IF1 [32, 47], which is overexpressed in LUAD (this study), as well as in breast and in colon carcinomas [48]. IF1 limits the ATP synthetic activity of the enzyme in cancer cells [47–49], as well as in normal mouse tissues in vivo [32, 50, 51], to favor the reprogramming of metabolism to an enhanced glycolytic phenotype. Interestingly, whereas the overexpression of IF1 in hepatocarcinomas [51, 52], gliomas, and in bladder and gastric carcinomas is pro-oncogenic (for review see [53]), multivariate analysis indicated that an increased content of IF1 in LUAD (this study), colon [31] and breast [54] carcinomas offers an independent marker of good prognosis, emphasizing the context dependency of some mitochondrial proteins in oncogenesis [55]. In agreement with previous studies in colon cancer cells with regulated expression of IF1 [31], we show that LUAD cells overexpressing IF1 have less in vitro and in vivo metastatic potential, supporting the finding that patients bearing LUAD with high levels of IF1 in their tumors have better prognosis.

Genomics has identified several oncogenic drivers and ICIs in LUAD that are exploited in targeted therapies [2, 3, 5]. However, the incorporation of metabolic enzymes in targeted therapies for LUAD patients is still waiting its birth. We suggest that new therapeutic opportunities are provided by some of the metabolic and redox enzymes described herein, especially those identified as independent prognostic markers that could be used to target the metabolic differences of LUAD. In this regard, GAPDH in glycolysis [56, 57] and OXPHOS [16–19, 23] have shown therapeutic efficacy restraining the growth of carcinomas. Of special relevance is the therapeutic potential offered by the repurposing of the FDA-approved β-adrenergic blocker nebivolol. Nebivolol, by binding to β1-adrenergic receptors that are expressed in tumor cells, causes the inhibition of OXPHOS at both Complex I and ATP synthase activities (Fig. 7) [18]. Moreover, nebivolol further arrests the proliferation of endothelial cells hampering tumor angiogenesis [18], what results in restricting the growth of colon, breast and squamous cell carcinomas by the induction of a metabolic and redox crisis [18, 38]. Similarly, we show here that nebivolol also arrests the growth of LUAD in vivo using a xenograft model. Although xenografts do not reproduce the lung microenvironment, which influences the metabolic dependencies of cancer cells [21, 22], they offer a valid model to assess the anticancer effect of drugs. In fact, we have previously demonstrated the potential of nebivolol to arrest tumor growth in an orthotopic model of colon cancer [18]. Altogether, these results strongly suggest that nebivolol is a strong candidate to be repurposed in cancer therapy.

We further highlight that inhibiting the oxidation of FA in mitochondria, either by blocking FA assimilation with orlistat, or by preventing FA import into mitochondria with etomoxir, effectively halt the growth of LUAD (Fig. 7). These findings are in agreement with results that targeted the pathway of FA oxidation at different levels, i.e., by pharmacologic inhibition of the trifunctional enzyme HADHA with trimetazidine [23] or by genetic ablation of long-chain fatty acyl-CoA synthetase (ACSL3), which is involved in the activation of FA [58]. Similar results have been obtained in leukemia cells [59], breast [60] and colon carcinomas [61], supporting the idea [36] that inhibitors of FA oxidation are promising drugs to be repurposed for the treatment of cancer in “basket” trials [62]. “Basket” trials are defined as those including cancer patients with carcinomas from different tissue origins but sharing a common mutation and/or biomarker.

Several inhibitors of FA oxidation have been approved for the treatment of human diseases [23, 36, 63]. However, over the years we have learned that targeting a single enzyme or pathway rarely cures cancer and hence, effective therapies need to combine different drugs to overcome the resistance of cancer cells to death. In this regard, we show that the combination of chemotherapies that target the mitochondrial electron transport chain and OXPHOS with the β1-adrenergic blocker nebivolol [18, 38], in combination with any of the drugs tested to prevent FA oxidation in mitochondria, could provide a new venue of hope for the effective and less noxious targeting of LUAD patients.

Overall, the changes in proteins of fatty acid oxidation, oxidative phosphorylation and of the antioxidant response are relevant diagnostic, prognostic and predictive markers of the disease, highlighting mitochondria as main targets of oncogenesis and metastasis and supporting their value for an effective and less toxic therapeutic option for LUAD.

## Materials and methods

### Human tissue specimens and preparation for processing

All lung tumors and NAT were obtained at the time of surgery at the Hospital Universitario Puerta de Hierro-Majadahonda (HUPH-M, Madrid, Spain) from January 2005 to February 2018. One hundred twenty-eight frozen tissue sections obtained from remnants of lung biopsies from patients diagnosed of LUAD were collected from the HUPH Biobank. A pathologist evaluated hematoxylin-stained cryostat sections used for protein isolation. The cohort consisted of sixty-nine LUAD and fifty-nine paired NAT of patients who had undergone radical resection without preoperative chemotherapy or radiotherapy. Patients’ medical records were reviewed, and identifiers coded to protect patient confidentiality. A summary of relevant clinicopathological information, including the maximum standardized uptake value (SUVmax) obtained in [18 F] FDG positron-emission tomography (PET) scans for some of the patients in the cohort, is provided in Supplementary Table S1. Frozen tissue sections were homogenized in Tissue Protein Extraction Reagent (T-PER) (Cat. No. 78510, Thermo Scientific, Ins. Madrid, Spain) supplemented with EDTA-free protease inhibitor cocktail (Complete Mini, MilliporeSigma, Burlington, Massachusetts, USA) and phosphatase inhibitor cocktail-2 (MilliporeSigma) in a 1:5 (w/v) ratio, and further freeze thawed three times in liquid nitrogen. Protein concentration was determined with Bradford reagent (Cat. No. 5000001,Bio-Rad, Hercules, California, USA).

### Reverse Phase Protein Array (RPPA)

The RPPA technique was used for quantification of steady-state protein levels in the biopsies as recently described in detail [64]. Only monospecific antibodies recognizing the expected protein in A549 cellular lysates were included in RPPA studies (Supplementary Fig. S1). Protein extracts from lung biopsies were diluted in PBS to a final protein concentration of 0.5 μg/μl before printing. Serially diluted protein extracts (0–1.0 μg/μl) of HCT116 and A549 cell lines, and of a tumor and NAT samples, were spotted onto the arrays (Supplementary Fig. S6). Printing quality and the linear response (Supplementary Fig. S7) of protein recognition by the antibodies used (Supplementary Table S3) was assessed. A standard curve of BSA (0–1 μg/μl) was also printed as internal negative control. Printing was carried out using a BioOdyssey Calligrapher MiniArrayer (Bio-Rad Laboratories, Inc. California, USA). Only 1 ng of protein extract from each sample was printed in duplicate onto nitrocellulose-coated glass slides (ONCYTE® SuperNOVA™ nitrocellulose film slides, Grace Bio-Labs, Inc. Oregon, USA) using a solid pin (MPC310S) at constant humidity (RH 45%) and temperature (16 °C). After printing, the slides were blocked for 1 h with Super G Blocking Buffer (Cat. No. 10501, Grace Bio-Labs, Inc. Madrid, Spain) and then incubated with the primary antibodies at the indicated dilutions (Supplementary Table S3) o/n at 4 °C. After incubation, the slides were washed with PBS-Tween 20 and further incubated with goat anti-mouse or anti-rabbit highly cross-absorbed secondary antibody conjugated with CF^TM^ 647 (Sigma Aldrich, SAB4600183–SAB4600185; 1:500; Madrid, Spain). Parallel pads were incubated with each of the secondary antibodies used to evaluate potential unspecific binding to non-masked human IgGs or with 0.0001% Fast Green FCF (Cat. No. F7252, Sigma Aldrich, Madrid, Spain), to evaluate the total protein amount present in the spotted samples. The mean fluorescent intensity of each spot was obtained by scanning microarrays with a Typhoon 9410 scanner (GE Healthcare, Inc. Madrid, Spain) and analyzed with GenePix® Pro 7 software (Molecular Devices, California, USA). The fluorescent intensity was normalized relative to the protein amount in the sample obtained from the FCF stained pad and further converted into arbitrary units of protein/ng of protein in the extract using as standard the linear plot of the HCT116 cell line (Supplementary Fig. S7) [64]. For RPPA analysis see Supplementary Methods.

### Metastasis assays and mouse xenograft studies

Male mice were excluded from the study because they are more aggressive and are less tolerant to treatments. For the in vivo studies, 6-week-old female athymic nude mice (NMRI-Foxn1nu, Envigo) with a body weight of 30–35 g were implanted with A549-Luc. In order to minimize the number of animals we used power analysis to calculate the minimum sample size using the free software DOEUMH (https://samplesizeumh.shinyapps.io/DOEUMH) based on the TrialSize library of the R program (R Core Team). We selected the procedure KMeans – ANOVA, fixing the significance to 0.05, power to 0.08 and a drop-out of 5%. We took into consideration differences between averages of about 1.5–2 fold. Minimum number of mice/group: 5–6 mice/group. Furthermore, the mice assays were not performed in a blinded fashion. For metastasis assays [33], nude mice were inoculated through the tail vein with 8 × 10^6^ A549-luc IF1 overexpressing or A549-luc IF1 silenced cells (8 mice per group). Randomization was assessed by equally distributing experimental groups across multiple cages and balancing the location of the mouse cages on the racks. Metastatic disease was monitored by in vivo imaging of the anesthetized mice after the i.p. administration of 150 mg kg^−1^ body weight of D-luciferin (Cat No. L2912, Promega. WI, USA) using the IVIS Lumina II equipment (Caliper Life Sciences. Massachusetts, USA) [31]. Animals were followed up during a maximum period of 4 weeks.

For xenograft studies, 6-week-old female nude mice with a body weight of 30–35 g were subcutaneously injected into the left and the right flanks with 4 × 10^6^ A549-Luc cells to develop LUAD. Tumor growth was monitored by bioluminescence as described above [18]. Tumor size was also determined using a standard caliper and its volume calculated using the formula (width^2^ x length) x 0.52, where width represents the shortest tumor dimension. When A549-Luc tumors reached ~100 mm^3^ of volume, animals were randomly allocated into different groups and were treated once a week with an i.p. injection of 5 mg kg^−1^ cisplatin (Cat.No, 232120 Sigma Aldrich, Madrid, Spain) or five days a week with a daily i.p. injection of the other compounds tested. Nebivolol (Cat. No. S1549 Selleckchem, Munich, Germany;10 mg kg^−1^), etomoxir (Cat. No. HY-50202, Bionova, Madrid, Spain; 40 mg kg^−1^) or orlistat (Cat. No. S1629, Selleckchem, Munich, Germany; 240 mg kg^−1^) in single or combined treatment were used as indicated. A 0.9 % NaCl treated group was included as a control. Mice were euthanized either 30 days after the initiation of treatment or following the ethical criteria established by our Institutional Review Board when the tumor volume reached ~ 1500 mm^3^. The tumor was removed for further analysis.

Other Methods are listed in Supplementary Methods.

## Supplementary information

Supplemental Material

## Data Availability

All data supporting the findings of this study are available within the article and its supplementary information files and from the corresponding author upon reasonable request.
