# Supplementary material for: Analysis of the metabolic proteome of lung adenocarcinomas by reverse-phase protein arrays (RPPA) emphasizes mitochondria as targets for therapy
Source: Oncogenesis. 2022 May 9;11(1):24. doi: 10.1038/s41389-022-00400-y (PMC9085865; doi:10.1038/s41389-022-00400-y)
Supplement: Supplementary file 1 — Supplemental Material [file 41389_2022_400_MOESM1_ESM.pdf]

Supplementary Materials and Methods

Cell culture and generation of cell lines. Cells were cultured in a humidified incubator at 37ºC with a controlled atmosphere of ambient air plus 5% CO_2. In order to maintain the optimal culture conditions for each cellular type human adenocarcinoma A549-Luc (ATCC® CCL-185-LUC2) (the parental wild-type and the engineered shIF1 and IF1) and H1975 (ATCC® CRL-5908) cells were grown in DMEM 4.5 g L^{-1} glucose media supplemented with 10% fetal bovine serum (FBS). The human colon cancer HCT116 cells (ATTC CCL-247) were cultured in McCoy's 5A 2 g L^{-1} glucose media plus 10% FBS. The human lung adenocarcinoma PC9 (provided by Dr. Luis Paz-Ares, Hospital Universitario 12 de Octubre, Madrid) and PC9-KO cells were cultured in RPMI 2 g L^{-1} glucose with 10% FBS. The generation of stable A549-luc ATPase Inhibitory Factor 1 (IF1) overexpressing IF1 (IF1) or IF1-silenced (shIF1) cells was carried out using lentivirus as recently described in detail (1). For overexpression, the pCDH-CMV-MCS-EF1-RFP+Puro cDNA Cloning and Expression Vector (SBI#CD616B-2; SBI System Biosciences. California, USA) was used (1). For IF1 silencing, we used the pEco-Lenti-U6 shRNA-(RFP-puro) (#LTSH-U6_RP, GenTarget Inc, California, USA.) silencer plasmid encoding the IF1 shRNA3 (Invitrogen, Thermo Fisher Scientific. Massachusetts, USA) driven by the U6 promoter (1). Viral particles were produced in HEK293T cells cultured in DMEM 10% FBS. After viral A549 transfection, stable transfectants were selected by adding 6 μg/ml puromycin (Invitrogen) to the growth medium. The generation of stable PC9 IF1-knock-out cells was carried out by the CRISPR-Cas9 technique. Briefly, we cloned into the pSpCas9(BB)-2A-GFP plasmid the IF1 guides (Fw: CACCGCAGTCCGAGAATGTCGACCG; Rv: AAACCGGTCGACATTCTCGGACTGC) in the BbsI site by Golden-gate cloning. Then, the cells were transfected with the plasmid using Lipofectamine 3000 (Thermo

Fisher Scientific, Madrid, Spain). Seventy-two hours after transfection, GFP+ cells were sorted (FACSAriaTM Fusion, BD Biosciences) for selection, and were expanded. All cell lines were tested as negative for mycoplasma contamination.

Cellular lysis and Western blotting. Cell lysis was performed with RLN-T buffer (RLN buffer plus 0.5% Triton X-100 and the complete protease and phosphatase inhibitor cocktails EDTA-free; Roche) at 20×10^6 cells/ml for 15 minutes on ice, freeze-thaw three times in liquid N_2 and clarified by centrifugation at 11,000 x g for 15 min at 4°C. Protein concentrations were determined with Bradford reagent using BSA as standard. The resulting supernatants were fractionated on SDS-6%, -9% or -12% PAGE and transferred onto PVDF or nitrocellulose membranes for immunoblot analysis using the primary antibodies and dilutions indicated in Supplemental Table S3 shown in Supplemental Figure S1. Peroxidase conjugated anti-mouse or anti-rabbit IgGs (Nordic Immunology, Rangeerweg, the Netherlands) were diluted in 5% non-fat-dried milk in TBS with 1% Tween 20 and used as secondary antibodies. The Novex® ECL (Cat. No. WP20005, Invitrogen. Madrid,Spain) system was used to visualize the bands. The intensity of the bands was quantified using a GS-900™ Calibrated Densitometer (Bio-Rad) and ImageJ Software.

RPPA data analysis. WEKA version 3.8 was used for a first inspection and visualization of data (2). Data processing was performed using Python language and Scikit-learn module version 0.20.3 (3). Raw data have been used in order to apply machine-learning methods. As the different markers distribution of each class are unimodal, the distance of each observation to their mode using Mahalanobis distance can be compute in order to identify possible outliers (4). Once the cubic root of the Mahalanobis distance of each observation to the covariance estimation is computed, interquartile range rule (IQR) was used to identify possible outliers and afterwards validated by hand. To exclude the

possible existence of some bias, outliers and missing values (less than the 1% of the data) were substituted by a random value generated using a robust covariance estimation and the trusted data of the sample (4). A t-test was applied to evaluate the equality of the means ($p < 0.05$). Normality of the biomarker's distribution being checked using normality test implemented in Scipy Python modules (5). Hierarchical clustering was performed using the Euclidean distance and Ward linkage methods implemented in Scipy Python modules (6). Pearson correlation matrix analysis and Principal Component Analysis (PCA) was applied to detect biomarker correlations and visualize class variability. Linear Discriminant Analysis (LDA) data transformation was carried out to obtain low-dimensional data that separates the two groups as much as possible, allowing also a graphical representation of the samples. Mostly all clinical data has been classified binarizing continuous values in different ranges. For each data classification, the most useful selection of biomarker combination was performed through two different processes. First, an algorithm has been developed so as to reject biomarkers non-suitable for the classification, then an exhaustive search was performed maximizing LDA classification score. LDA classifiers combined using One-Vs-One strategy was used as classification model. Leave-One-Out Cross-Validation procedure was applied, where statistical measures as sensitivity and specificity were computed besides Receiver Operating Characteristic (ROC) curves. Kaplan-Meir curves/analysis were performed with Lifelines v0.22.9 python module and compared by log-rank test. The Cox proportional hazard regression model was used in determining the value of independent prognostic factors.

Cellular O_2 consumption and glycolysis rates. Metabolic readouts of different cell lines were performed using substrates at the same concentration in order to maintain comparability. Oxygen consumption rates were determined in A549-Luc, H1975 and PC9

cell lines in a XF24 Extracellular Flux Analyzer (Agilent Technologies, California, USA) using 10 mM glucose, 1 mM pyruvate and 2 mM glutamine (7). For respiration using palmitate as substrate, cells were starved for 12 h in low glucose DMEM (0.05 mM glucose, 1% FBS), and then changed to KHB media (111 mM NaCl, 4.7 mM KCl, 1.25 mM glutamine, 5 mM HEPES, pH 7.4). BSA-conjugated palmitate (1 mM sodium palmitate, 0.17 mM BSA solution) was added as the main substrate. To assess oligomycin sensitive respiration, maximum respiration, and non-mitochondrial dependent oxygen consumption, respectively, 6 μM oligomycin (OL), 0.75 mM 2,4-dinitrophenol (DNP), and 1 μM rotenone plus 1 μM antimycin were added. The initial rates of lactate production were determined as previously described (8).

Mitochondrial membrane potential (ΔΨm) and ROS production. For ΔΨm assessment, transfected A549 and PC9 cells were treated with 100 nM JC1 (Molecular Probes. Madrid,Spain) and 100 nM TMRM (Invitrogen. Madrid,Spain) respectively and processed for flow cytometry (9). ROS production was determined using H_2DCFDA (dichlorofluorescin diacetate) (Thermo Fisher, Massachusetts, USA) by flow cytometry (9). The fluorescence intensity of at least 10,000 events was determined in a FACScan cytometer (BD Biosciences) and analyzed using the FLOWJO software (Tree Star, Oregon, USA).

Cellular proliferation, cell death and cell invasion assays. Cellular proliferation was determined by the incorporation of 5-ethynyl-20deoxy-uridine (EdU) into cellular DNA using the Click-iT EdU Flow Cytometry Assay Kit (Thermo Fisher, Massachusetts, USA) (1). For cell death assays, 50,000 cells/well were seeded and treated or not with hydrogen peroxide (H_2O_2) during 24h. Cell death was determined by flow cytometry after staining with Annexin V (ApoScreen FITC; SouthernBiotech. Alabama, USA)(1) . Corning Biocoat Matrigel Invasion Chambers (Cat.No. DLW354480. Merck. Madrid,Spain) (8.0

μm pore size) were used to quantify the cellular invasive capacity (1). A total of 2×10^4 cells were seeded in 1% FBS and chemoattraction perform during 72 h in 20% FBS.

RNA isolation and RT-PCR analysis. RNA was isolated from A459 cells with RNeasy Mini Kit (Qiagen, Hilden, Germany), according to the manufacturer's instructions. Purified RNA was quantified with a Nanodrop Sspectrophotometer (Thermo Fisher Scientific), and 1.5 μg was retrotranscribed into cDNA with the High-Capacity cDNA Reverse Transcription Kit (Thermo Fisher Scientific). Real-time PCR was performed with Fast SYBR Master Mix in an ABI PRISM 7900HT sequence detection system (Thermo Fisher Scientific) from the Genomics and NGS Core Facility (CBMSO, Madrid, Spain). Thermal cycling conditions were as follows: initial denaturation of 20 s at 95 ºC, 40 amplification cycles of 1 s at 95 ºC and 20 s at 60 ºC each, followed by a dissociation curve analysis to detect possible non-specific amplification. Standard curves with serial dilutions of pooled cDNA were used to assess amplification efficiency of some primers and to establish the dynamic range of cDNA concentration for amplification, which was 3 ng of input RNA per run. The relative expression of the mRNAs was determined with the comparative $\Delta\Delta C_T$ method with β-actin and 18S as controls. Primers used to amplify the target genes are indicated in Supplementary Table S4. Pre-designed primers were acquired from MilliporeSigma (KiCqStart™ SYBR® Green Primers).

Tumor analysis. Lung tumors were fixed in 4% paraformaldehyde (Merck) and included in OCT blocks. Frozen 15-μm sections were stained with hematoxylin/eosin or incubated with anti-α smooth muscle Actin-Cy3 (Cat. No.C6198.1:100, Sigma Aldrich) to assess angiogenesis, anti-cleaved caspase-3 (Cat.No.9661,1:200, Cell Signaling, Madrid, Spain) to assess cell death and anti-Ki67 (1:250, Thermo Fisher Scientific) to assess proliferation. Nuclei were counter stained with DAPI (diamidino-2-fenilindol) reagent. Cellular fluorescence was analyzed by confocal microscopy in an Olympus SpinSR10

and Nikon A1R+ microscopes. The area and intensity of fluorescence were quantified by ImageJ v1.46 software.

Other statistical analysis. The results shown are the means ± SEM. Statistical analysis were performed by Student's t-test and/or one-way ANOVA with the Tukey's or Dunnett's multiple comparisons test. Statistical tests were two-sided at the 5% level of significance. Graphics and statistical analyses were performed using GraphPad Prism 7.

Supplemental Information

Supplemental Table S1. Clinicopathological characteristics of tumor (T) biopsies in the cohort of LUAD patients

**Samples**	**No. Patients**
Non-tumor	59
Tumor	69
**Male/Female**	52/17
**Size**	
≤3	28
≥3-7	41
**Pleural Invasion**	
Yes	33
No	33
Unknown	3
**Vascular Invasion**	
Yes	33
No	33
Unknown	3
**Stage**	
I	40
II/III/V	25
non-available	4
**SUVmax**	
< 7.5	25
≥ 7.5	19
non-available	25

Supplemental Table S2. Transcriptomic analysis of shIF1, wild-type and IF1 A549 cells. The table summarizes the relative expression of the genes determined in A549 cells, as fold of wild-type. The results shown are the mean values ± SEM. χ $P < 0.05$ and $\chi\,\chi$ $P < 0.01$ with respect to wild-type; ϕ $P < 0.05$ and $\phi\,\phi$ $P < 0.01$ with respect to shIF1 by one-way ANOVA and Tukey's multiple comparisons tests.

**Category**	**Gene**	**shIF1**	**wt**	**IF1**
**Mitochondria**	PDHB	0.75 ± 0.03	1.00 ± 0.06	2.51 ± 0.29$^{\chi\chi\,\theta\theta}$
	GLS	0.94 ± 0.17	1.00 ± 0.15	1.16 ± 0.05
	GLUD1	0.66 ± 0.15	1.00 ± 0.26	4.81 ± 0.56$^{\chi\chi\,\theta\theta}$
	CS	1.43 ± 0.04	1.00 ± 0.11	2.05 ± 0.24$^{\chi\chi}$
	IDH3A	1.12 ± 0.08	1.00 ± 0.07	1.26 ± 0.14
	MDH2	0.77 ± 0.32	1.00 ± 0.23	2.51 ± 0.15$^{\chi\,\theta\theta}$
	NDUFA1	1.39 ± 0.01	1.00 ± 0.09	4.61 ± 0.56$^{\chi\chi\,\theta\theta}$
	ND6	1.86 ± 0.52	1.00 ± 0.08	1.83 ± 0.36
	COX7B	1.00 ± 0.02	1.00 ± 0.09	0.75 ± 0.07
	ATP5F1	0.83 ± 0.15	1.00 ± 0.14	2.93 ± 0.15$^{\chi\chi\,\theta\theta}$
	β-F1	1.34 ± 0.04	1.00 ± 0.12	1.79 ± 0.18$^{\chi\chi}$
	IF1	0.68 ± 0.09	1.00 ± 0.08	4.54 ± 0.45$^{\chi\chi\,\theta\theta}$
	TFAM	1.04 ± 0.06	1.00 ± 0.16	2.25 ± 0.18$^{\chi\chi\,\theta\theta}$
	MTERF	1.25 ± 0.05	1.00 ± 0.13	1.75 ± 0.15$^{\chi\chi}$
	TUFM	1.10 ± 0.02	1.00 ± 0.22	1.46 ± 0.04
	HSP60	1.14 ± 0.07	1.00 ± 0.07	1.39 ± 0.02$^{\chi\chi\,\theta}$
	ALAS1	1.03 ± 0.10	1.00 ± 0.14	3.52 ± 0.44$^{\chi\chi\,\theta\theta}$
	CYB5B	0.60 ± 0.02$^{\chi}$	1.00 ± 0.14	0.51 ± 0.05$^{\chi}$
**Glycolysis**	HK2	0.14 ± 0.01$^{\chi\chi}$	1.00 ± 0.13	9.47 ± 0.20$^{\chi\chi\,\theta\theta}$
	GAPDH	1.29 ± 0.03$^{\chi}$	1.00 ± 0.07	0.55 ± 0.01$^{\chi\chi\,\theta\theta}$
	ENO1	0.27 ± 0.07	1.00 ± 0.29	2.13 ± 0.41$^{\theta\theta}$
	LDHA	0.73 ± 0.02	1.00 ± 0.09	0.68 ± 0.05$^{\chi}$
**Antioxidant system**	SOD2	0.36 ± 0.07	1.00 ± 0.09	2.24 ± 0.29$^{\chi\chi\,\theta\theta}$
	CAT	0.58 ± 0.06$^{\chi\chi}$	1.00 ± 0.05	0.97 ± 0.06$^{\theta\theta}$
	PRDX6	1.17 ± 0.02	1.00 ± 0.06	1.55 ± 0.18$^{\chi}$
	GSR	0.84 ± 0.18	1.00 ± 0.27	0.96 ± 0.04
	TXN	1.61 ± 0.07$^{\chi\chi}$	1.00 ± 0.09	0.77 ± 0.06$^{\theta\theta}$
**Cell cycle**	CDK4	1.26 ± 0.06	1.00 ± 0.11	1.57 ± 0.13$^{\chi}$
	CDKN1B	1.59 ± 0.08$^{\chi}$	1.00 ± 0.14	2.40 ± 0.11$^{\chi\chi\,\theta\theta}$
	RB1	0.95 ± 0.23	1.00 ± 0.30	3.67 ± 0.28$^{\chi\chi\,\theta\theta}$
	TP53	1.19 ± 0.21	1.00 ± 0.24	1.47 ± 0.08
	MTBP	1.78 ± 0.20	1.00 ± 0.02	3.39 ± 0.41$^{\chi\chi\,\theta}$
**Cell death and survival**	BCLXL	1.00 ± 0.04	1.00 ± 0.16	0.14 ± 0.01$^{\chi\chi\,\theta\theta}$
	BNIP3	0.62 ± 0.09	1.00 ± 0.19	0.32 ± 0.03$^{\chi}$
	AIFM1	1.35 ± 0.03	1.00 ± 0.14	2.03 ± 0.23$^{\chi\chi\,\theta}$
	DDIT3	1.78 ± 0.05$^{\chi\chi}$	1.00 ± 0.14	0.69 ± 0.02$^{\theta\theta}$
**Cell signaling and transcription factors**	VEGFA	0.93 ± 0.11	1.00 ± 0.06	0.71 ± 0.06
	ADM	2.08 ± 0.14$^{\chi\chi}$	1.00 ± 0.17	0.43 ± 0.04$^{\theta\theta}$
	ANGPTL4	2.07 ± 0.20$^{\chi}$	1.00 ± 0.22	0.00 ± 0.00$^{\chi\,\theta\theta}$
	CXCR4	0.13 ± 0.01	1.00 ± 0.04	6.00 ± 1.21$^{\chi\chi\,\theta\theta}$
	SEMA5A	2.22 ± 0.57	1.00 ± 0.58	0.87 ± 0.18

	TGFB1	1.21 ± 0.13	1.00 ± 0.10	0.85 ± 0.08
	TGFB3	0.96 ± 0.08	1.00 ± 0.19	52.7 ± 10.7$^{\chi\chi\,\theta\theta}$
	SMAD3	1.39 ± 0.30	1.00 ± 0.10	0.13 ± 0.03$^{\chi\,\theta\theta}$
	CTNNB1	1.01 ± 0.01	1.00 ± 0.09	2.81 ± 0.17$^{\chi\chi\,\theta\theta}$
	GSK3B	1.14 ± 0.04	1.00 ± 0.11	0.94 ± 0.04
	NOTCH1	2.51 ± 0.31$^{\chi\chi}$	1.00 ± 0.12	3.14 ± 0.15$^{\chi\chi}$
	JAG1	1.03 ± 0.02	1.00 ± 0.16	0.39 ± 0.03$^{\chi\chi\,\theta\theta}$
	JAG2	0.93 ± 0.11	1.00 ± 0.13	4.37 ± 0.30$^{\chi\chi\,\theta\theta}$
	GPS2	0.91 ± 0.05	1.00 ± 0.09	0.87 ± 0.03
	ATF4	1.75 ± 0.20	1.00 ± 0.05	4.76 ± 0.58$^{\chi\chi\,\theta\theta}$
	MET	0.86 ± 0.16	1.00 ± 0.10	0.13 ± 0.01$^{\chi\chi\,\theta\theta}$
	FOS	1.37 ± 0.13	1.00 ± 0.07	19.3 ± 1.7$^{\chi\chi\,\theta\theta}$
	JUN	1.60 ± 0.12	1.00 ± 0.15	4.03 ± 0.16$^{\chi\chi\,\theta\theta}$
	MYC	1.24 ± 0.09	1.00 ± 0.13	2.18 ± 0.20$^{\chi\chi\,\theta}$
	HES1	0.77 ± 0.02	1.00 ± 0.11	6.47 ± 0.10$^{\chi\chi\,\theta\theta}$
	HEY1	4.28 ± 0.48	1.00 ± 0.14	493 ± 24$^{\chi\chi\,\theta\theta}$
	POU5F1B	1.88 ± 0.28$^{\chi}$	1.00 ± 0.09	1.23 ± 0.04
	RUNX2	0.27 ± 0.02$^{\chi\chi}$	1.00 ± 0.08	0.32 ± 0.03$^{\chi\chi}$
**Cell adhesion and extracellular matrix**	ITGB1	0.64 ± 0.06$^{\chi}$	1.00 ± 0.10	0.13 ± 0.02$^{\chi\chi\,\theta\theta}$
	THBS1	3.13 ± 1.13	1.00 ± 0.18	0.00 ± 0.00$^{\theta}$
	SERPINB7	1.23 ± 0.32	1.00 ± 0.26	0.46 ± 0.15
	SERPINB11	0.83 ± 0.12	1.00 ± 0.48	1.29 ± 0.53
	VCAN	0.06 ± 0.01$^{\chi\chi}$	1.00 ± 0.16	0.51 ± 0.09$^{\chi}$
	CYR61	1.25 ± 0.15	1.00 ± 0.13	0.18 ± 0.01$^{\chi\chi\,\theta\theta}$
**Miscellaneous**	GJA1	0.56 ± 0.08	1.00 ± 0.10	0.73 ± 0.14
	ACTA2	0.38 ± 0.12	1.00 ± 0.25	0.39 ± 0.15
	F3	0.66 ± 0.05	1.00 ± 0.07	1.05 ± 0.15
	CIB1	1.69 ± 0.15	1.00 ± 0.50	0.87 ± 0.03
	LCN2	0.10 ± 0.01$^{\chi\chi}$	1.00 ± 0.16	0.00 ± 0.00$^{\chi\chi}$
	UPP1	1.00 ± 0.04	1.00 ± 0.13	0.15 ± 0.01$^{\chi\chi\,\theta\theta}$
	IGF2BP1	1.50 ± 0.09	1.00 ± 0.18	2.23 ± 0.26$^{\chi\chi}$
	LC3	0.94 ± 0.05	1.00 ± 0.12	2.35 ± 0.17$^{\chi\chi\,\theta\theta}$
	ATG3	0.98 ± 0.06	1.00 ± 0.13	1.51 ± 0.03$^{\chi\,\theta\theta}$
	NPM1	1.41 ± 0.16	1.00 ± 0.25	1.71 ± 0.88
	PHGDH	3.97 ± 0.10	1.00 ± 0.03	30.3 ± 3.5$^{\chi\chi\,\theta\theta}$

Supplemental Table S3. **Source and dilutions of the specific antibodies used**.

NAME	Provider	Reference	DILUTION WB	DILUTION RPPA
**GAPDH**	homemade	(10)	1:1000	1:250
**ENO1**	Abgent	AM219b	1:500	1:250
**LDHA**	homemade	(11)	1:1000	1:100
**GPD1**	homemade	(11)	1:1000	1:150
**MPC1**	Cell Signaling	144625	1:500	1:100
**PDH-E1α**	Invitrogen	459400	1:500	1:50
**SDH-B**	Invitrogen	#459230	1:500	1:200
**MDH2**	Proteintech	15462-1-AP	1:1000	1:100
**CPT1**	Santa Cruz	sc-20670	1:200	1:100
**ETFa**	Abcam	ab110316	1:1000	1:100
**HADHA**	Abcam	ab54477	1:500	1:100
**ACYL**	Abcam	ab40793	1:1000	1:100
**FAS**	BD transduction Laboratories	610962	1:1000	1:100
**Hsp60**	homemade	(10)	1:1000	1:250
**CORE II**	Abcam	ab14745	1:1000	1:100
**β-F1 ATPase**	homemade	(10)	1:1000	1:100
**IF1**	homemade	(8)	1:1000	1:100
**G6PDH**	Thermo Scientific	#PA1-84814	1:1000	1:100
**GR**	Abcam	ab59543	1:1000	1:250
**SOD1**	Santa Cruz	sc-11407	1:1000	1:100
**CATALASE**	Sigma Aldrich	C0979	1:10000	1:200
**PRX2**	Abcam	ab109367	1:1000	1:250
**PRX6**	Abcam	ab59543	1:1000	1:250
**THIOREDOXIN**	Cusabio	PA02814A0Rb	1:1000	1:100
**SOD2**	Abcam	13533	1:1000	1:100
**PRX3**	Abcam	ab222807	1:1000	1:250
**IDH1**	Millipore	ABE454	1:1000	1:200
**MDA**	Abcam	ab27642	1:1000	1:250
**4-HNE**	RD Systems	MAB3249	1:1000	1:200
**NitTyr**	Abcam	ab110282	1:1000	1:250
**Flip**	Cell Signaling	#8510S	1:500	
**Bxl-xL**	Cell Signaling	#2764	1:1000	
**Integrin -β1**	Abcam	Ab30394	1:1000	

**β-Catenin**	BD Bioscience	610154	1:500	
**α-Tubulin**	Sigma	T9026	1:20000	
**E-cadherin**	BD Bioscience	610181	1:500	

Supplemental Table S4. Primers used for RT-PCR analysis of A549 cells.

Target gene	Sequence or reference
PDHB	F 5'-CGGATAGAGGACACGACCA-3' and R 5'-TTATAGCATCACGAACTGTCACC-3'
GLS	Sigma, Gene ID 2744, primer pair 1
GLUD1	F 5'-CATTCATGTATTGTCCAGCAGAC-3' and R 5'-GTAAGCAAATTCTGACAGTTTTAATGA-3'
CS	F 5'-GCATCTTGTCTTGTTCTTGCAG-3' and R 5'-TGGCCTGCTCCTTAGGTATC-3'
IDH3A	F 5'-TGGGGTTGCAATTTTTGAGT-3' and R 5'-GGATTCGCCATGTCCTTG-3'
MDH2	Sigma, Gene ID 4191, primer pair 1
NDUFA1	F 5'-GCAGTTTCTGGCACATGGTA-3' and R 5'-AAATGTAAGTTTCTTCCTGGGCTA -3'
ND6	F 5'-GGGGTTAGCGATGGAGGTAG-3' and R 5'-CCAATAGGATCCTCCCGAAT-3'
COX7B	F 5'-AGCGCACTAAATCGTCTCCA-3' and R 5'-GAAAATCAGGTGTACGTTTCTGG-3'
ATP5F1	Sigma, Gene ID 515, primer pair 1
β-F1	F 5'-CTTCAATGGGTCCCACCATA-3' and R 5'-CAGCAGATTTTGGCAGGTG-3'
IF1	F 5'-GGGCCTTCGGAAAGAGAG-3' and R 5'-TTCAAAGCTGCCAGTTGTTC-3'
TFAM	F 5'-ATATTTAAAGCTCAGAACCCAGATG-3' and R 5'-GAATCAGGAAGTTCCCTCCA-3'
MTERF	F 5'-GGCGGAAGTAAAAGCGAAC-3' and R 5'-TAAGGAAAGGCTCTGCATCC-3'
TUFM	F 5'-ATGTGGACCACGGGAAGAC-3' and R 5'-GGCATTGTCAATCTCCTCGTA-3'
HSP60	F 5'- TGCTATGGCTGGAGATTTTGT-3' and R 5'-CAGCAGCATCCAATAAAGCA-3'
ALAS1	F 5'-CAGTAATGACTACCTAGGAATGAGTCG-3' and R 5'-CCATGTTGTTTCAAAGTGTCCA-3'
CYB5B	F 5'-GGCGAGTCTACGATGTCACC-3' and R 5'-GCTTGTTCCAGCAGAACCTC-3'
HK2	F 5'-TCCCCTGCCACCAGACTA-3' and R 5'-TGGACTTGAATCCCTTGGTC-3'
GAPDH	F 5'-AGCCACATCGCTCAGACAC-3' and R 5'-GCCCAATACGACCAAATCC-3'
ENO1	Sigma, Gene ID 2023, primer pair 1
LDHA	F 5′-TCTCTGTAGCAGATTTGGCAGA-3' and R 5′-AAGACATCATCCTTTATTCCGTAAA-3'
SOD2	Sigma, Gene ID 6648, primer pair 1
CAT	Sigma, Gene ID 847, primer pair 2
PRDX6	F 5'-GGACGTGGCTCCCAACTTT-3' and R 5'-CGAGGGTGGGAGAAGAGAATG-3'
GSR	Sigma, Gene ID 2936, primer pair 1
TXN	F 5'-AAGTCAAATGCATGCCAACATTC-3' and R 5'-AATGGTGGCTTCAAGCTTTTCCT-3'
CDK4	F 5'-GAGGAGTCGGGAGCACAG-3' and R 5'-CTCCGGATTACCTTCATCCTT-3'

CDKN1B	F 5’-CCCTAGAGGGCAAGTACGAGT-3’ and R 5’-AGTAGAACTCGGGCAAGCTG-3’
RB1	F 5’-AATTGGAAAGGACATGTGAACTTAT-3’and R 5’-CCAATGCAGAATTTATTTCAGTAGATA-3’
TP53	Sigma, Gene ID 7157, primer pair 1
MTBP	Sigma, Gene ID 27085, primer pair 3
BCLXL	F 5’-AGCCTTGGATCCAGGAGAA-3’ and R 5’-AGCGGTTGAAGCGTTCCT-3’
BNIP3	F 5’-GAATTTCTGAAAGTTTTCCTTCCA-3’ and R 5’-TTGTCAGACGCCTTCCAAT-3’
AIFM1	F 5’-CAAGCACGCTCTAACATCTGG-3’ and R 5’-GCGTGATCATGGTGCTCTAC-3’
DDIT3	F 5’-CAGAGCTGGAACCTGAGGAG-3’ and R 5’-CTGCAGTTGGATCAGTCTGG-3’
VEGFA	F 5’-AGTGTGTGCCCACTGAGGA-3’ and R 5’-GGTGAGGTTTGATCCGCATA-3’
ADM	Sigma, Gene ID 133, primer pair 1
ANGPTL4	Sigma, Gene ID 51129, primer pair 1
CXCR4	Sigma, Gene ID 7852, primer pair 2
SEMA5A	Sigma, Gene ID 9037, primer pair 2
TGFB1	Sigma, Gene ID 7040, primer pair 2
TGFB3	Sigma, Gene ID 7043, primer pair 2
SMAD3	Sigma, Gene ID 4088, primer pair 3
CTNNB1	F 5’-GAATCCATTCTGGTGCCACT-3’ and R 5’-TCCTCAGGATTGCCTTTACC-3’
GSK3B	F 5’-GAAAGTATTGCAGGACAAGAGATTT-3’ and R 5’-CGGACTATGTTACAGTGATCTAGCTT-3’
NOTCH1	F 5’-CCTGCTGCCCTACACAGG-3’ and R 5’-AGCTCTCATAGTCCTCGGATTG-3’
JAG1	F 5’-GAATGGCAACAAAACTTGCAT-3’ and R 5’-AGCCTTGTCGGCAAATAGC-3’
JAG2	F 5’-TCATCCCCTTCCAGTTCG-3’ and R 5’-ATGCGACACTCGCTCGAT-3’
GPS2	Sigma, Gene ID 2874, primer pair 1
ATF4	F 5′-TCTCCAGCGACAAGGCTAA-3’ and R 5′-CAATCTGTCCCGGAGAAGG-3’
MET	Sigma, Gene ID 4233, primer pair 3
FOS	F 5’-CTACCACTCACCCGCAGACT-3’ and R 5’-AGGTCCGTGCAGAAGTCCT-3’
JUN	F 5’-CCAAAGGATAGTGCGATGTTT-3’ and R 5’-CTGTCCCTCTCCACTGCAAC-3’
MYC	F 5’-AAAAAGCCACAGCATACATCC-3’ and R 5’-CCGCAACAAGTCCTCTTCA-3’
HES1	F 5’-GAAGCACCTCCGGAACCT-3’ and R 5’-GTCACCTCGTTCATGCACTC-3’
HEY1	F 5’-TATCGGAGTTTGGGATTTCG-3’ and R 5’-GCATCTAGTCCTTCAATGATGCT-3’
POU5F1B	F 5’-AATCTTCAGGAGATATGCAAAGCA-3’ and R 5’-CTCGGTTCTCGATACTGGTTCG-3’
RUNX2	F 5’-CCCGTCCATCCACTCTACC-3’ and R 5’-GCCTAGGCACATCGGTGA-3’
ITGB1	Sigma, Gene ID 3688, primer pair 3
THBS1	Sigma, Gene ID 7057, primer pair 1

SERPINB7	Sigma, Gene ID 8710, primer pair 2
SERPINB11	Sigma, Gene ID 89778, primer pair 1
VCAN	Sigma, Gene ID 1462, primer pair 3
CYR61	Sigma, Gene ID 3491, primer pair 1
GJA1	Sigma, Gene ID 2697, primer pair 1
ACTA2	Sigma, Gene ID 59, primer pair 3
F3	Sigma, Gene ID 2152, primer pair 2
CIB1	Sigma, Gene ID 10519, primer pair 1
LCN2	F 5'-CAGGACTCCACCTCAGACCT-3' and R 5'-CACATACCACTTCCCCTGGA-3'
UPP1	F 5'-AGGACACTGCCTGGAACG-3' and R 5'-TTCAGCTTTCTCTGCATTGG-3'
IGF2BP1	F 5'-AAGTTCGGGAGGCCTATGAG-3' and R 5'-CAGGGATCAGGTGAGACTGC-3'
LC3	F 5'-CGCACCTTCGAACAAAGAG-3' and R 5'-CTCACCCTTGTATCGTTCTATTATCA-3'
ATG3	F 5'-AGGTATTACAGGAATAACGGAAGC-3' and R 5'-CAATCTTGAAGCCTTATATTGTCCT-3'
NPM1	F 5'-GAATGGAAAAGACTCAAAACCATCA-3' and R 5'-ACTTTGGGAAGAGAACCACCTTT-3'
PHGDH	F 5'-CAAGGAAGGGCATCTTGGT-3' and R 5'-TCATTCCACAAGTGAGTTCTGC-3'
β-actin	F 5'-CCAACCGCGAGAAGATGA-3' and R 5'-CCAGAGGCGTACAGGGATAG-3'
18S	F 5'-GCAATTATTCCCCATGAACG-3' and R 5'-GGGACTTAATCAACGCAAGC-3'

Supplemental Table S5. Comparison of patients' prognosis in LUAD using metabolic proteins as biomarkers in proteomic and transcriptomic studies. Prognostic significance of the comparison of absolute RPPA values, or Z-score normalized expression of the biomarkers, with transcriptomic studies of the selected genes using the TGCA-Pancancer Atlas and the GDC TCGA databases. The transcriptomic results show no relevant differences in prognosis for seven of the biomarkers studied, the same prediction as in our study for GAPDH, β-F1-ATPase and SOD2, and just the opposite prediction for patients' prognosis for the overexpression of HSP60. The table also incorporates studies that support that the expression of these biomarkers correlate with prognosis of the patients.

		PROTEOMIC						TRANSCRIPTOMIC		
		RPPA (this study)			IHC/WB			RNA-seq		
	Levels	p value Absolute	p value Z-score	Prognosis	Cancer	Ref.	Prognosis	Pancancer p value	GDC p value	Prognosis
**GAPDH**	High	0.006	0.01	Bad	Breast, Melanoma	(12, 13)	Bad	0.004	0.02	Bad
**GPD1**	High	0.01	0.04	Good	-	-	-	n.s	n.s	-
**CPT1**	High	0.01	0.06	Good	-	-	-	n.s	n.s	-
**ETFA**	High	0.008	0.03	Good	-	-	-	n.s	n.s	-
**HADHA**	High	0.001	0.04	Good	Renal cell carcinoma	(14, 15)	Good	n.s	n.s	-
**Hsp60**	High	0.001	0.004	Good	Hepatocellular carcinoma	(16)	Good	0.01	0.03	Bad
					Breast, Melanoma	(12, 13)	Bad			
**β-F1**	High	0.001	0.004	Bad	Breast	(12)	Bad	0.02	0.001	Bad
					Colon, Leukemia	(17-19)	Good			
**IF1**	High	0.0005	0.008	Good	Breast, colon	(1, 20)	Good	n.s	n.s	-
					Glioma, Bladder, Gastric, Lung, Hepatocellular carcinoma	(21-26)	Bad			
**PRX3**	High	0.04	0.2	Bad	Melanoma	(27)	Bad	n.s	n.s	-
**SOD2**	High	0.02	0.4	Bad	Lung, Colon, Prostate	(28)	Bad	0.03	n.s	Bad
**PRX6**	High	0.0001	0.0001	Good	Prostate, Lymphoma	(29, 30)	Bad	n.s	n.s	-

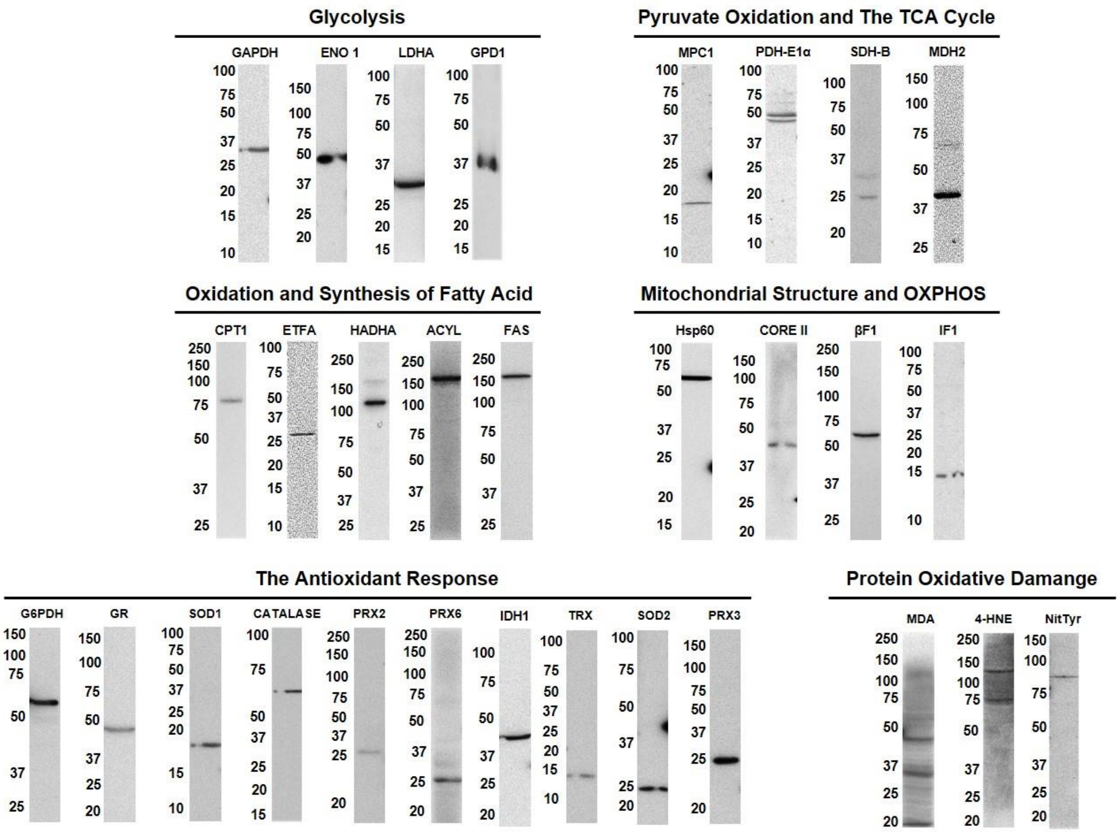

Supplemental Figure S1. Validation of the antibodies used in RPPA. Extracts (30 µg of protein) derived from A549 human lung cancer cells were fractionated on SDS-PAGE and blotted to check the specificity of the antibodies used in this study. Only antibodies that recognize a single protein band of the expected molecular mass, with the exception of markers of oxidative damage (MDA, 4-HNE, Nitrotyrosine), were used in the study. The migration of molecular mass markers (kDa) is indicated to the left.

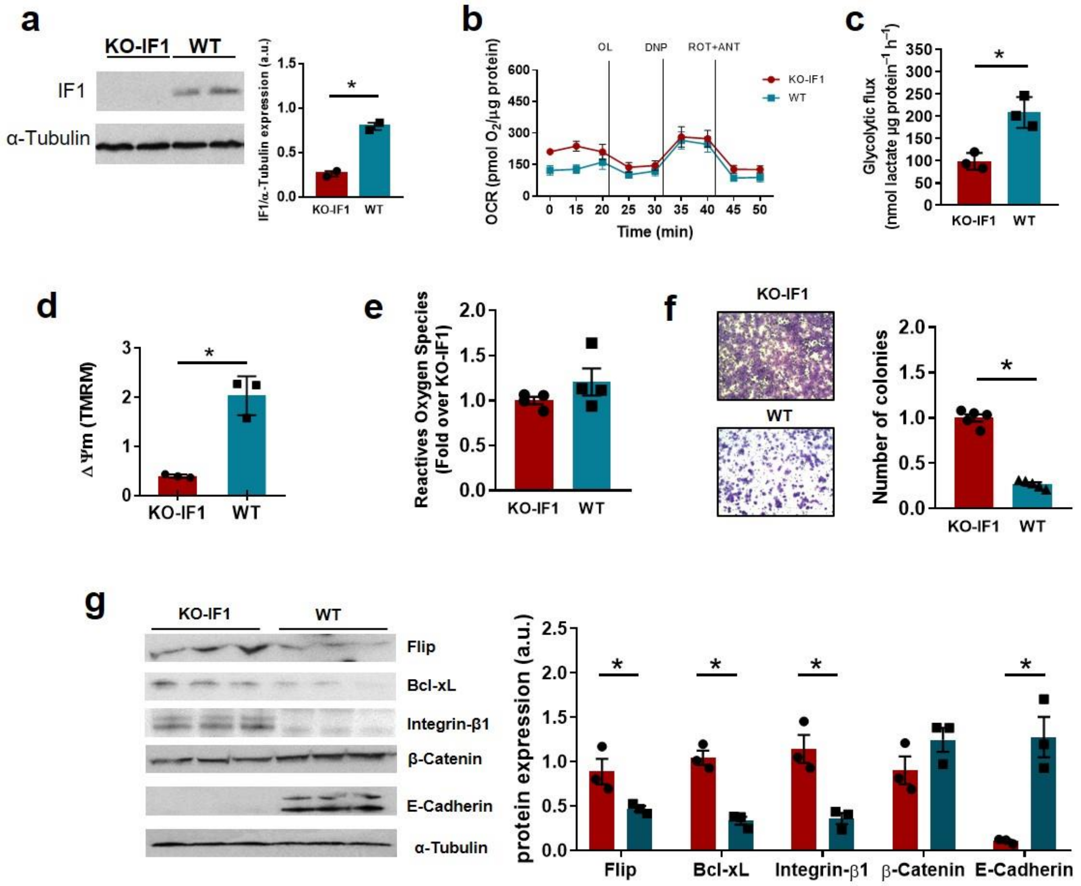

Supplemental Figure S2. IF1 knockout PC-9 cells have an invasive phenotype. IF1 knockout PC-9 (KO-IF1, red bars) cells were developed by the CRISPR-Cas9 technique and compared to parental (WT, blue bars) PC-9 cells. **a)** Quantification of IF1 expression (n=2). Respiratory profile (n=6) **b)** and glycolytic flux (n=3) **c)** of the cells. **d)** Mitochondrial membrane potential (ΔΨm) using TMRM is expressed as fold change of WT (n=3). **e)** ROS production using H_2DCFDA fluorescence is expressed as fold change of WT (n=4). **f)** Representative images of the Matrigel invasion assays of KO-IF1 (upper) and WT (lower) cells at 72 h. The histogram shows the invasion normalized to shIF1 cells (n=5). **j)** Expression of *anoikis*, apoptosis, extracellular matrix and epithelial–mesenchymal transition proteins in three different replicates of KO-IF1 and WT cells (n=3). Tubulin is used as loading control. Results shown are means ±S.E.M; *, p≤0.05 by Student's t test.

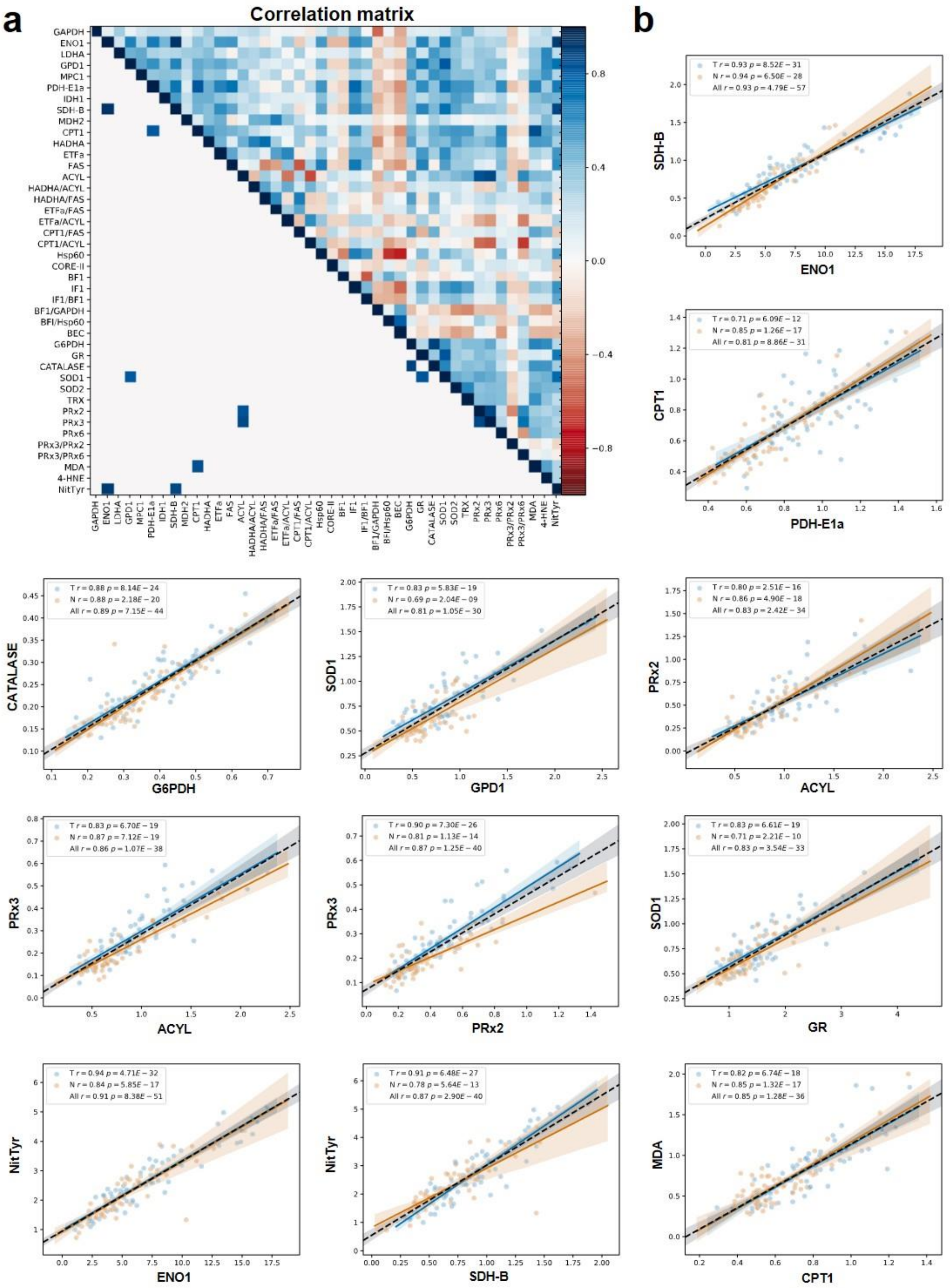

Supplemental Figure S3. Correlations between protein expression levels in lung samples. a) Pearson correlation matrix for NAT (N, n=59) and tumor (T, n=69) samples quantified by RPPA shows the R2 of Huber loss linear regression values of pairwise marker comparison of the individual expression levels for the thirty markers and ratios studied. Correlation scores between markers are shown according to a color scale, from -

1 (red) to +1 (blue). In the bottom-left side of the plot, a threshold of +0.8 was applied on the R2 correlation score, where values below are marked as white. **b)** Scatter plots showing significant correlations between proteins of energy metabolism in N (orange) and T (blue) samples with a R2 correlation score > +0.8. r- and *p*-values for N, T and All (N+T) classes of samples are also indicated.

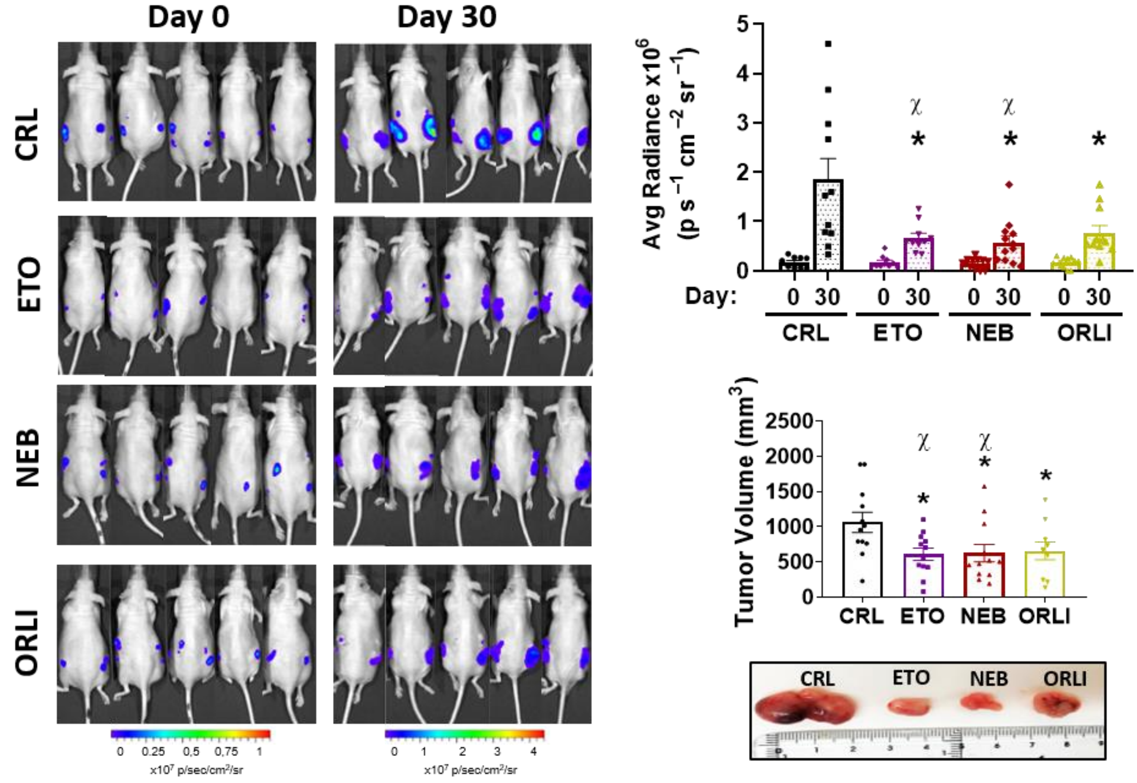

Supplemental Figure S4. Growth of lung adenocarcinomas requires the efficient oxidization of fatty acids. A549-Luc cells were injected into the flanks of nude mice. When tumors reached a volume of ~ 100 mm^3, mice were treated with saline (CRL; black bar; n = 6), 40 mg kg^{-1} etomoxir (ETO; purple bar; n=6), 10 mg kg^{-1} nebivolol (NEB; red bar; n = 6) or 240 mg kg^{-1} orlistat (ORLI, yellow bar; n = 6). Mice were euthanized 30 days after treatment initiation to verify the effect of the drugs in tumor growth. Left panel shows representative images of the bioluminescence of A549-Luc tumors in mice at day 0 and after day 30 of treatments initiation. Right upper panel, quantification of light emission of the implanted cells (CRL, n = 12; ETO, n=12; NEB n =12; ORLI n=12). Lower panel, quantification of tumor volume (mm^3) after 30 days of treatment (CRL, n = 12; ETO, n=12; NEB n =12; ORLI n=12). Bars indicate the mean of indicated tumors ± SEM. *, $p<0.05$ when compared to CRL by two-sided Student's t test. χ $p < 0.05$ when compared to CRL by one-way ANOVA and Dunnett's multiple comparisons tests. Representative images of tumors are also shown.

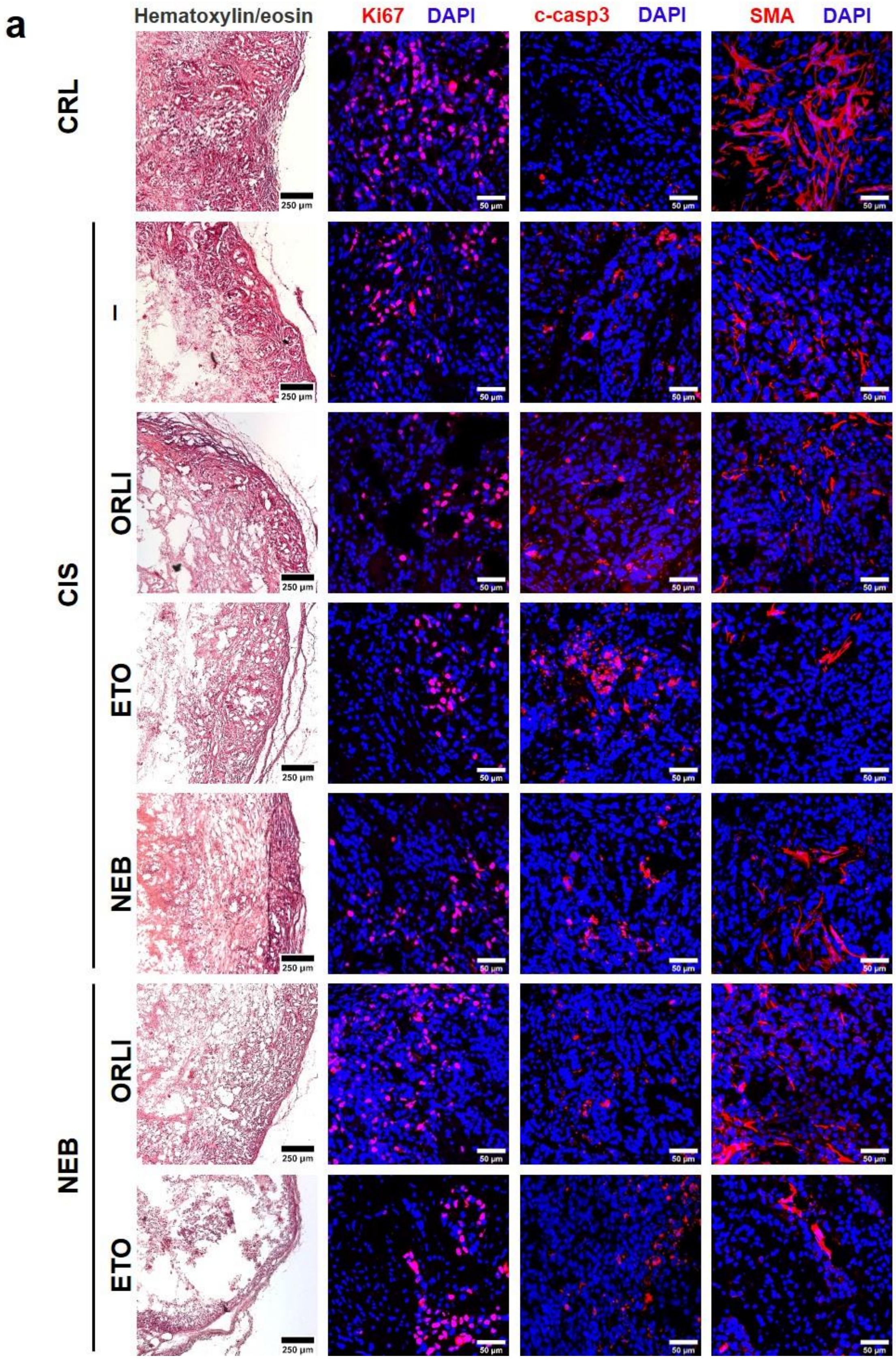
a
Hematoxylin/eosin
Ki67 DAPI
c-casp3 DAPI
SMA DAPI
CRL
CIS
–
ORLI
ETO
NEB
NEB
ORLI
ETO
250 µm
50 µm

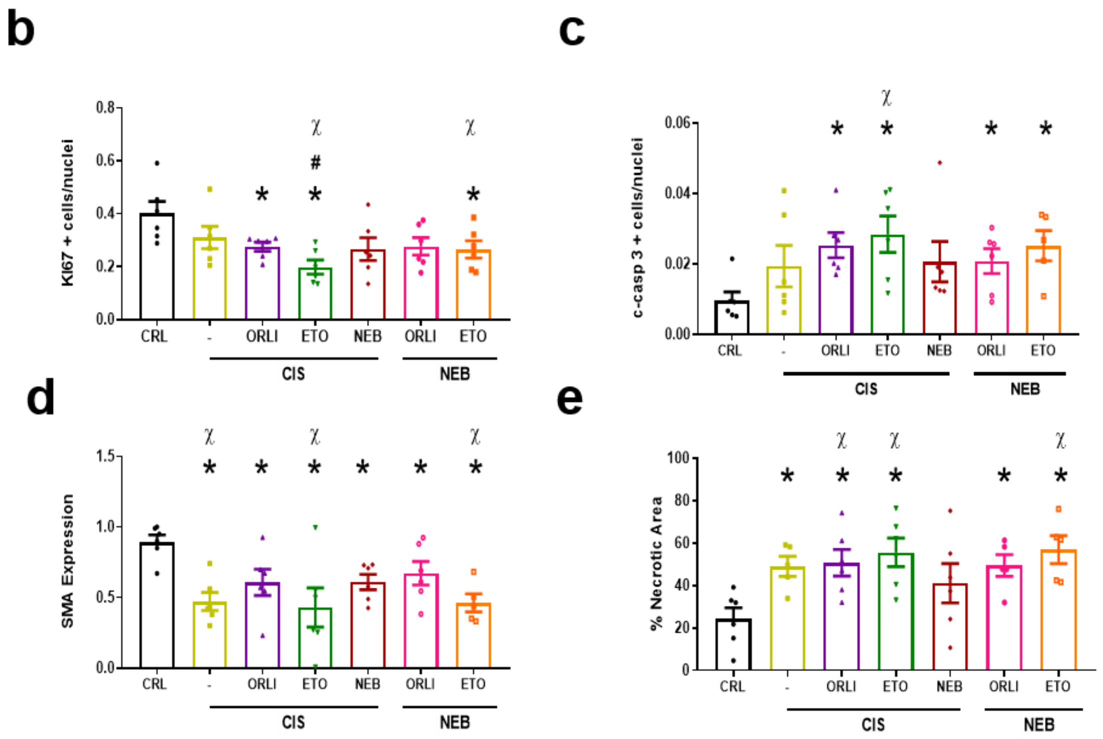

Supplemental Figure. S5. Effect of treatments in proliferation, cell-death, angiogenesis and necrosis of the carcinomas. a-e) A549-Luc carcinomas obtained from euthanized mice from saline (CRL; black bars; n = 6), 5 mg kg^{-1} cisplatin (CIS, yellow bars; n = 6); 5 mg kg^{-1} cisplatin plus 240 mg kg^{-1} orlistat (CIS+ORLI, purple bars; n = 6); 5 mg kg^{-1} cisplatin plus 40 mg kg^{-1} etomoxir (CIS+ETO; green bars; n=6), 5 mg kg^{-1} cisplatin plus 10 mg kg^{-1} nebivolol (CIS+NEB, red bars; n = 6), 10 mg kg^{-1} nebivolol plus 240 mg kg^{-1} orlistat (NEB+ORLI, pink bars; n= 6) or 10 mg kg^{-1} nebivolol plus 40 mg kg^{-1} etomoxir (NEB+ETO, orange bars; n=6) were hematoxylin/eosin stained (scale bars, 250 µm) or processed by immunofluorescence microscopy to reveal Ki67, cleaved active caspase-3 (c-casp3) or αSMA (in red). Nuclei were DAPI (blue)-stained. Scale bars, 50 µm. Histograms in **b-c**) show the rates of cellular proliferation (**b**, Ki67+ cells) and cell-death (**c**, c-casp 3 + cells) relative to cell nuclei. Histograms in **d-e)** show the angiogenesis (**d**, SMA expression/tumor area) and percentage of necrosis **e**) of the carcinomas. Bars indicate the mean of six different biological samples ± SEM. * and #, $p < 0.05$ when compared to CRL or CIS by two-sided Student's t test, respectively. χ $p < 0.05$ when compared to CRL by one-way ANOVA and Dunnett's multiple comparisons tests.

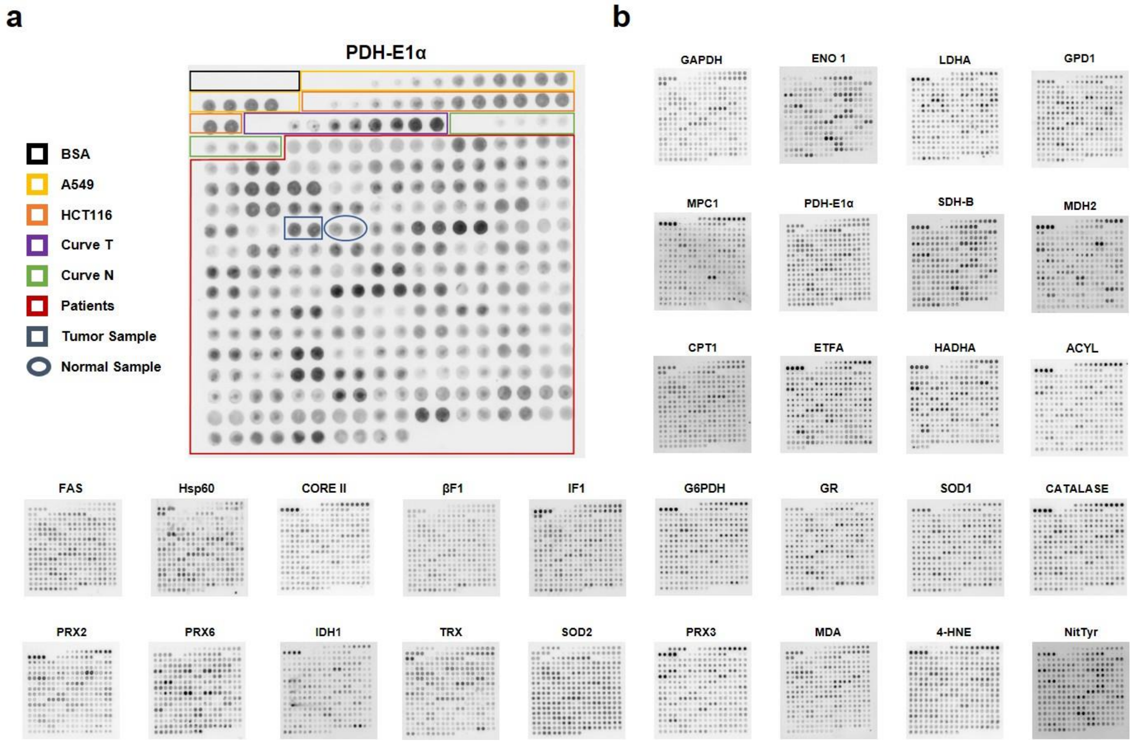

Supplemental Figure S6. Printing of RPPA. a) The scheme of RPPA printing processed for anti-PDH-E1α is shown magnified for details. One nl samples were spotted in duplicate. *Black boxed*, negative controls of BSA; *Yellow boxed*, standard curves of A549 cells; *Orange boxed*, standard curves of HCT116 cells; *Purple boxed*, standard curves of tumor sample (T); *Green boxed*, standard curves of NAT sample (N); *Red boxed*, area of T (blue rectangle) and N (blue ellipse) samples; **b)** Representative RPPAs processed with other antibodies.

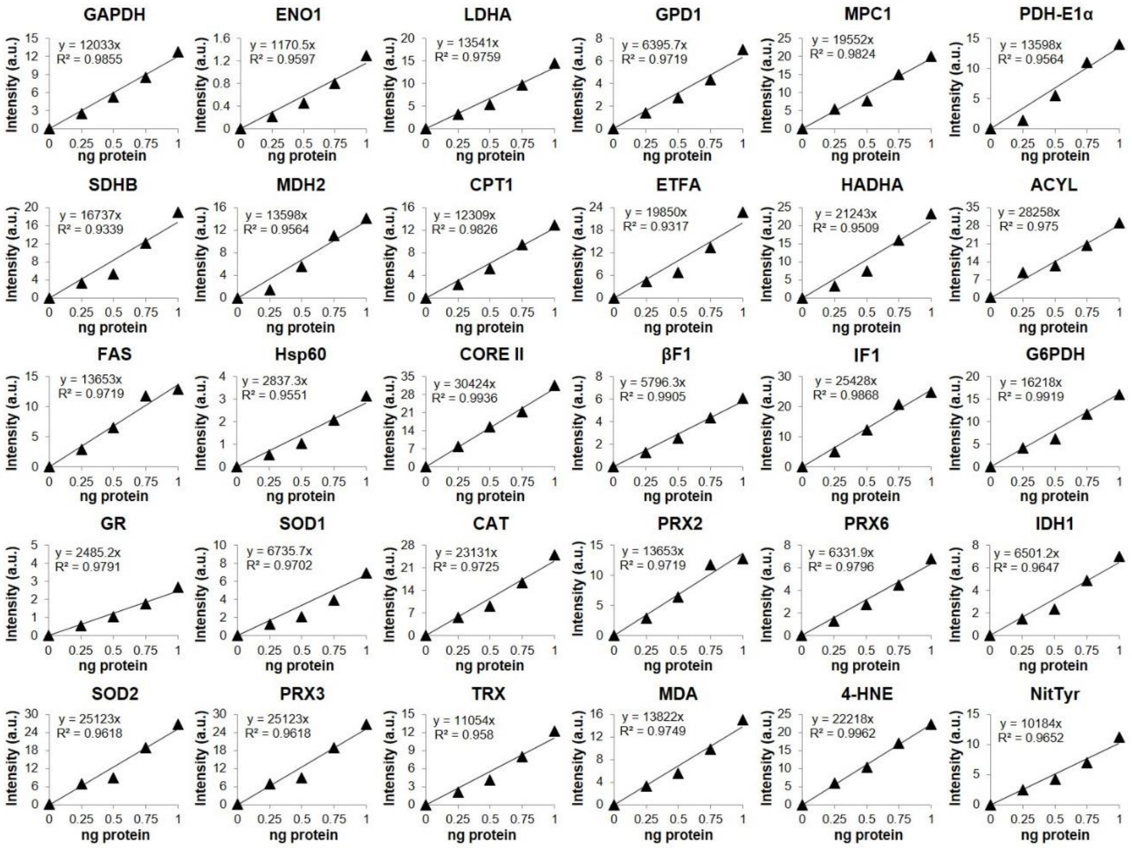

Supplemental Figure. S7. Linear correlations between the fluorescence intensity signals and the amount of printed proteins. HCT116 cell line extracts (0–1 μg/μl) were spotted into the arrays (see Supplemental Fig. S2). Significant linear correlations were obtained between the fluorescence intensity signals (arbitrary units, a.u.) of the spots and the amount of protein in the arrays. Protein concentrations in the biopsies were calculated by interpolation in the respective linear plots.

References

1. Gonzalez-Llorente L, Santacatterina F, Garcia-Aguilar A, Nuevo-Tapioles C, Gonzalez-Garcia S, Tirpakova Z, et al. Overexpression of Mitochondrial IF1 Prevents Metastatic Disease of Colorectal Cancer by Enhancing Anoikis and Tumor Infiltration of NK Cells. Cancers (Basel). 2019;12.
2. Witten IH, Frank E, Hall MA, Pal CJ. Data Mining: Practical machine learning tools and techniques: Morgan Kaufmann; 2016.
3. Pedregosa F, Varoquaux G, Gramfort A, Michel V, Thirion B, Grisel O, et al. Scikit-learn: Machine learning in Python. Journal of Machine Learning Research. 2011;12:2825-30.
4. Peña D, Prieto FJ. Multivariate Outlier Detection and Robust Covariance Matrix Estimation. Technometrics. 2001;43:286-310.
5. Virtanen P, Gommers R, Oliphant TE, Haberland M, Reddy T, Cournapeau D, et al. SciPy 1.0: fundamental algorithms for scientific computing in Python. Nature methods. 2020;17:261-72.
6. Jones E, Oliphant T, Peterson P. {SciPy}: open source scientific tools for {Python}. 2014.
7. Nuevo-Tapioles C, Santacatterina F, Stamatakis K, Nunez de Arenas C, Gomez de Cedron M, Formentini L, et al. Coordinate beta-adrenergic inhibition of mitochondrial activity and angiogenesis arrest tumor growth. Nat Commun. 2020;11:3606.
8. Sanchez-Cenizo L, Formentini L, Aldea M, Ortega AD, Garcia-Huerta P, Sanchez-Arago M, et al. Up-regulation of the ATPase inhibitory factor 1 (IF1) of the mitochondrial H+-ATP synthase in human tumors mediates the metabolic shift of cancer cells to a Warburg phenotype. J Biol Chem. 2010;285:25308-13.
9. Formentini L, Sánchez-Aragó M, Sánchez-Cenizo L, Cuezva JM. The mitochondrial ATPase Inhibitory Factor 1 (IF1) triggers a ROS-mediated retrograde pro-survival and proliferative response. Mol Cell. 2012;45:731-42.
10. Acebo P, Giner D, Calvo P, Blanco-Rivero A, Ortega AD, Fernandez PL, et al. Cancer abolishes the tissue type-specific differences in the phenotype of energetic metabolism. Transl Oncol. 2009;2:138-45.
11. Santacatterina F, Chamorro M, Nuñez de Arenas C, Navarro C, Martin MA, Cuezva JM, et al. Quantitative analysis of proteins of metabolism by reverse phase protein microarrays identifies potential biomarkers of rare neuromuscular diseases. J Trans Med. 2015;13:65.
12. Isidoro A, Casado E, Redondo A, Acebo P, Espinosa E, Alonso AM, et al. Breast carcinomas fulfill the Warburg hypothesis and provide metabolic markers of cancer prognosis. Carcinogenesis. 2005;26:2095-104.
13. Najera L, Alonso-Juarranz M, Garrido M, Ballestin C, Moya L, Martinez-Diaz M, et al. Prognostic implications of markers of the metabolic phenotype in human cutaneous melanoma. Br J Dermatol. 2019;181:114-27.
14. Zhao Z, Lu J, Han L, Wang X, Man Q, Liu S. Prognostic significance of two lipid metabolism enzymes, HADHA and ACAT2, in clear cell renal cell carcinoma. Tumour Biol. 2016;37:8121-30.

15. Jiang H, Chen H, Wan P, Chen N. Decreased expression of HADH is related to poor prognosis and immune infiltration in kidney renal clear cell carcinoma. Genomics. 2021;113:3556-64.

16. Zhang J, Zhou X, Chang H, Huang X, Guo X, Du X, et al. Hsp60 exerts a tumor suppressor function by inducing cell differentiation and inhibiting invasion in hepatocellular carcinoma. Oncotarget. 2016;7:68976-89.

17. Cuezva JM, Krajewska M, de Heredia ML, Krajewski S, Santamaria G, Kim H, et al. The bioenergetic signature of cancer: a marker of tumor progression. Cancer Res. 2002;62:6674-81.

18. Lin PC, Lin JK, Yang SH, Wang HS, Li AF, Chang SC. Expression of beta-F1-ATPase and mitochondrial transcription factor A and the change in mitochondrial DNA content in colorectal cancer: clinical data analysis and evidence from an in vitro study. Int J Colorectal Dis. 2008;23:1223-32.

19. Xiao X, Yang J, Li R, Liu S, Xu Y, Zheng W, et al. Deregulation of mitochondrial ATPsyn-beta in acute myeloid leukemia cells and with increased drug resistance. PLoS One. 2013;8:e83610.

20. Garcia-Ledo L, Nuevo-Tapioles C, Cuevas-Martin C, Martinez-Reyes I, Soldevilla B, Gonzalez-Llorente L, et al. Overexpression of the ATPase Inhibitory Factor 1 Favors a Non-metastatic Phenotype in Breast Cancer. Front Oncol. 2017;7:69.

21. Wu J, Shan Q, Li P, Wu Y, Xie J, Wang X. ATPase inhibitory factor 1 is a potential prognostic marker for the migration and invasion of glioma. Oncol Lett. 2015;10:2075-80.

22. Wei S, Fukuhara H, Kawada C, Kurabayashi A, Furihata M, Ogura S, et al. Silencing of ATPase Inhibitory Factor 1 Inhibits Cell Growth via Cell Cycle Arrest in Bladder Cancer. Pathobiology. 2015;82:224-32.

23. Yin T, Lu L, Xiong Z, Wei S, Cui D. ATPase inhibitory factor 1 is a prognostic marker and contributes to proliferation and invasion of human gastric cancer cells. Biomed Pharmacother. 2015;70:90-6.

24. Gao YX, Chen L, Hu XG, Wu HB, Cui YH, Zhang X, et al. ATPase inhibitory factor 1 expression is an independent prognostic factor in non-small cell lung cancer. Am J Cancer Res. 2016;6:1141-8.

25. Song R, Song H, Liang Y, Yin D, Zhang H, Zheng T, et al. Reciprocal activation between ATPase inhibitory factor 1 and NF-kappaB drives hepatocellular carcinoma angiogenesis and metastasis. Hepatology. 2014;60:1659-73.

26. Santacatterina F, Sanchez-Cenizo L, Formentini L, Mobasher MA, Casas E, Rueda CB, et al. Down-regulation of oxidative phosphorylation in the liver by expression of the ATPase inhibitory factor 1 induces a tumor-promoter metabolic state. Oncotarget. 2016;7:490-508.

27. Ramasamy P, Larkin AM, Linge A, Tiernan D, McAree F, Horgan N, et al. PRDX3 is associated with metastasis and poor survival in uveal melanoma. J Clin Pathol. 2020;73:408-12.

28. Miar A, Hevia D, Munoz-Cimadevilla H, Astudillo A, Velasco J, Sainz RM, et al. Manganese superoxide dismutase (SOD2/MnSOD)/catalase and SOD2/GPx1 ratios as

biomarkers for tumor progression and metastasis in prostate, colon, and lung cancer. Free Radic Biol Med. 2015;85:45-55.

29. Kuusisto ME, Haapasaari KM, Turpeenniemi-Hujanen T, Jantunen E, Soini Y, Peroja P, et al. High intensity of cytoplasmic peroxiredoxin VI expression is associated with adverse outcome in diffuse large B-cell lymphoma independently of International Prognostic Index. J Clin Pathol. 2015;68:552-6.

30. Raatikainen S, Aaaltomaa S, Karja V, Soini Y. Increased Peroxiredoxin 6 Expression Predicts Biochemical Recurrence in Prostate Cancer Patients After Radical Prostatectomy. Anticancer Res. 2015;35:6465-70.
